# Supplementary material for: Nonlinear optical diode effect in a magnetic Weyl semimetal
Source: Nat Commun. 2024 Apr 8;15:3017. doi: 10.1038/s41467-024-47291-8 (PMC11271640; doi:10.1038/s41467-024-47291-8)
Supplement: Supplementary file 1 — Supplementary Information [file 41467_2024_47291_MOESM1_ESM.pdf]

Supplementary Information

Nonlinear optical diode effect in a magnetic Weyl semimetal

C. Tzschaschel *et al.*

CONTENTS

1. Materials and Methods 3
   A. Symmetry considerations in CeAlSi 3
   B. Electrode patterning 4
   C. SHG tensor components of CeAlSi 5
   D. SHG interference phenomena 5

2. Polarization-resolved SHG measurements 10

3. Temperature dependent characterization 12

4. Additional NODE spectroscopy 14
   A. Observation of a broadband NODE in the $\mathbf{M}_{-\mathrm{y}}$ state 14
   B. Determination and robustness of directional contrast 14
   C. Directional contrast in the $\mathbf{M}_{\pm\mathrm{x}}$ states 15
   D. A note on symmetry 17
   E. Directional contrast in the paramagnetic phase 18

5. Domain assignment and mapping 19

6. Magnetic field dependent SHG characterization 21

7. Symmetry of the NODE 23

8. Integrated SHG spectrum 25

9. FIB sample 27

10. First-principles calculations 30
A. Electronic structure of CeAlSi 30
B. k-space resolved χ contributions 34

References 36

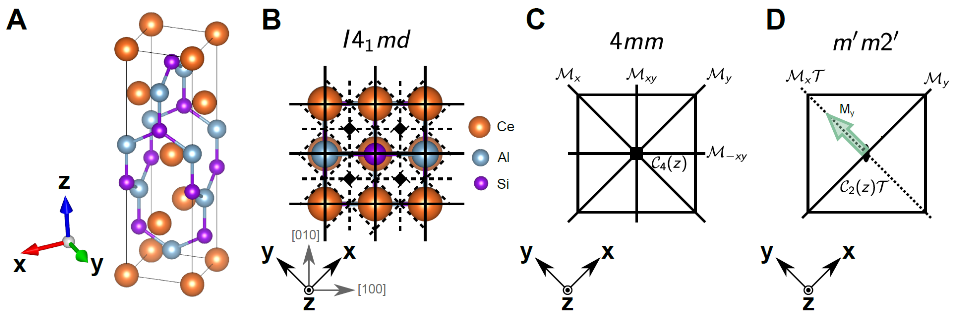

Supplementary Fig. 1. **Lattice structure and symmetry of CeAlSi. a,** 3D representation of the paramagnetic unit cell of CeAlSi (point group $4mm$, space group #109: $I4_1md$) **b,** Projection of the the unit cell along $\hat{\mathbf{z}}$ axis. Solid lines, dashed lines and diamonds indicate the positions of mirror planes, glide-mirror planes and screw rotational axis, respectively. Here, we use an xyz coordinate system that is 45° rotated with respect to the conventional tetragonal unit cell (grey). **c,** Illustration of point group symmetries of the paramagnetic point group $4mm$ projected along $\hat{\mathbf{z}}$ axis. **d,** Illustration of point group symmetries of the ferromagnetic point group $m'm2'$ projected along $\hat{\mathbf{z}}$ axis. The magnetization M_y along the $\hat{\mathbf{y}}$ axis reduces the symmetry from $4mm$ to $m'm2'$.

1. MATERIALS AND METHODS

A. Symmetry considerations in CeAlSi

Throughout the Supplementary Material, all Miller indices $[hkl]$ are given with respect to the conventional tetragonal unit cell of the paramagnetic phase (Fig. 1). The directions $\hat{\mathbf{x}}$, $\hat{\mathbf{y}}$, $\hat{\mathbf{z}}$ are defined as $[110] = +\hat{\mathbf{x}}$, $[\bar{1}10] = +\hat{\mathbf{y}}$, and $[001] = +\hat{\mathbf{z}}$.

In this section, we describe the symmetry of the paramagnetic and ferromagnetic phases of CeAlSi. The symmetry dictates the nonzero components of the SHG tensor χ_{ijk} as defined by

$$P_i(2\omega) = \chi_{ijk}E_j(\omega)E_k(\omega). \tag{1}$$

The paramagnetic phase of CeAlSi crystallizes in the same tetragonal lattice as TaAs, with the space group #109 ($I4_1md$) and the point group $4mm$ (Fig. 1A). Just like in the case of TaAs, all atoms occupy sites belonging to a 4a-Wyckoff orbit. Table 1 enumerates the symmetry operations of the paramagnetic phase of CeAlSi.

Paramagnetic CeAlSi	$\mathcal{E}$	$\mathcal{C}_4(z)$	$\mathcal{C}_2(z)$	$\mathcal{M}_x$	$\mathcal{M}_y$	$\mathcal{M}_{xy}$	$\mathcal{M}_{-xy}$
	$\mathcal{T}$	$\mathcal{C}_4(z)\mathcal{T}$	$\mathcal{C}_2(z)\mathcal{T}$	$\mathcal{M}_x\mathcal{T}$	$\mathcal{M}_y\mathcal{T}$	$\mathcal{M}_{xy}\mathcal{T}$	$\mathcal{M}_{-xy}\mathcal{T}$

Supplementary Table 1. **Point group symmetry of the paramagnetic phase of CeAlSi** ($4mm$).

$\mathcal{E}$ is identity, $\mathcal{T}$ is time-reversal symmetry, $\mathcal{C}_4(z)$ and $\mathcal{C}_2(z)$ are the 4-fold rotation and 2-fold rotation around the $\hat{\mathbf{z}}$ axis, and $\mathcal{M}_i$ represents the mirror reflection from i to $-i$ ($i = x, y, xy, -xy$). Because the system has time-reversal

symmetry $\mathcal{T}$, any spatial symmetry (mirror, rotation, etc) combined with $\mathcal{T}$ is also a good symmetry, as reflected in the second line above.

The ferromagnetic phase of CeAlSi exhibits a spontaneous magnetization along $\hat{\mathbf{x}}$ or $\hat{\mathbf{y}}$. The magnetization breaks not only time-reversal symmetry $\mathcal{T}$ but also a number of other symmetries. As a result, the point group symmetry is reduced to $m'm2'$. Supplementary Table 2 shows the remaining magnetic symmetry operations in the ferromagnetic phase for a domain with $\boldsymbol{M}^{\mathrm{Ce}} \parallel \hat{\mathbf{y}}$ [1].

Ferromagnetic CeAlSi	$\mathcal{E}$	$\mathcal{M}_y$
	$\mathcal{C}_2(z)\mathcal{T}$	$\mathcal{M}_x\mathcal{T}$

Supplementary Table 2. **Point group symmetry of the ferromagnetic phase of CeAlSi** ($m'm2'$) **with** $\boldsymbol{M} \parallel \hat{\mathbf{y}}$ **[1].**

B. Electrode patterning

In order to apply current in the crystallographic xy plane, we deposited electrical contacts on a naturally cleaved (001) facet (see Fig. 2). A square pattern of eight gold electrodes was deposited at room temperature by e-beam evaporation through a shadow mask. Aligning the shadow mask with a naturally cleaved edge allows us to apply current along the crystallographic $\langle 110\rangle$ or $\langle 100\rangle$ direction, which corresponds to perpendicular/parallel or at an angle of 45° relative to the magnetization direction in CeAlSi, respectively. Subsequently, the crystal was glued and wire bonded onto a chip carrier. Current was applied using a Keithley Model 2400 SourceMeter.

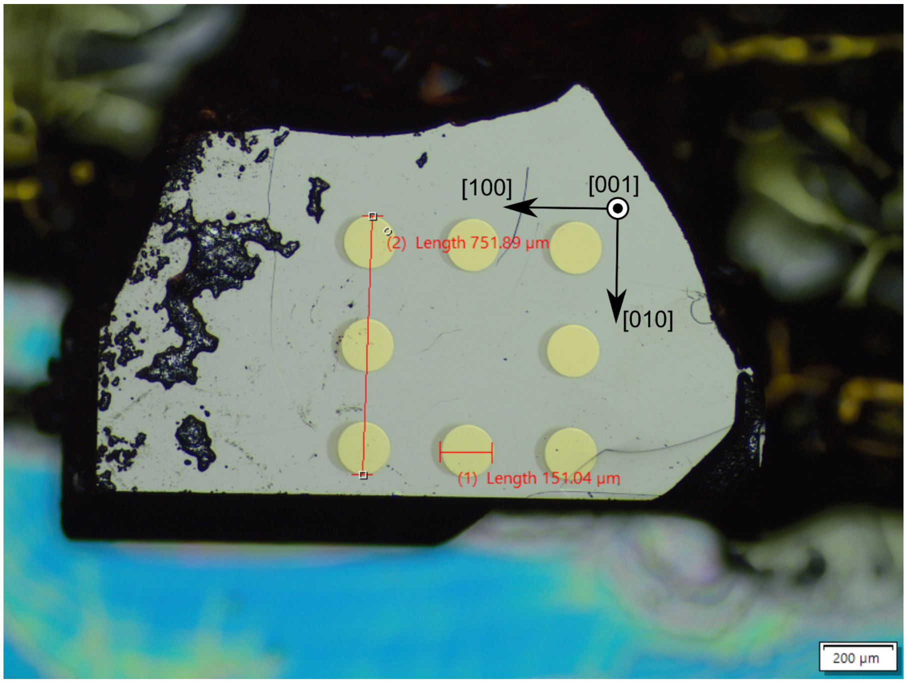

Supplementary Fig. 2. **Optical microscope image of CeAlSi device with electrode pattern.**

C. SHG tensor components of CeAlSi

The symmetry of the paramagnetic phase of CeAlSi has the point group $4mm$. A symmetry analysis similar to the previous section shows that the allowed components of the SHG tensor introduced in Eq. (1) are [1]

$$\chi_{zzz} \qquad \chi_{zxx} = \chi_{zyy} \qquad \chi_{xxz} = \chi_{xzx} = \chi_{yyz} = \chi_{yzy}. \tag{2}$$

All other tensor components vanish. Specifically, no electric-dipole SHG is allowed for light propagating along the z direction.

Below T_{C}, the magnetic order reduces the symmetry to $Fd'd2'$ (#43.3.322, point group $m'm2'$) [2], which gives rise to additional contributions to the second-order susceptibility. In particular, the allowed components below T_{C} are (for a magnetization along the y direction):

$$\chi_{zzz} \qquad \chi_{zxx} \qquad \chi_{zyy} \qquad \chi_{xxz} = \chi_{xzx} \qquad \chi_{yyz} = \chi_{yzy} \tag{3}$$

$$\chi_{xxx} \qquad \chi_{xyy} \qquad \chi_{xzz} \qquad \chi_{zzx} = \chi_{zxz} \qquad \chi_{yyx} = \chi_{yxy}. \tag{4}$$

Note that the allowed χ tensor components for the four magnetic states of CeAlSi are related by symmetry as shown in Supplementary Table 3.

The polarization dependence of the measured SHG intensity can be simulated as

$$I_{2\omega} \propto |\boldsymbol{P}(2\omega) \cdot \boldsymbol{A}|^2, \tag{5}$$

where $\boldsymbol{P}(2\omega)$ is the second-order polarization as described by Eq. (1) and $\boldsymbol{A}$ is a unit-vector defining the transmission axis of the analyzer crystal for the SHG light. All tensor components are in general complex quantities, i.e. $\chi_{ijk} = |\chi_{ijk}| \exp{(i\Phi_{ijk})}$.

D. SHG interference phenomena

Interference between the various contributions to the nonlinear optical polarization $\boldsymbol{P}(2\omega)$ can give rise to several intriguing phenomena including the nonlinear optical diode effect described in the main text.

In the following we distinguish in particular between three separate, but sometimes overlapping, effects: magnetic domain contrast, nonreciprocal SHG (NR-SHG), and the nonlinear optical diode effect (NODE). We illustrate their relative relationship in a Venn diagram in Fig. 3

1. **Magnetic domain contrast** describes a difference in SHG intensity between different magnetic domains. A magnetic domain contrast can be observed within the electric-dipole approximation. This is due to interference

	$\mathbf{M}_{+\mathrm{y}}$	$\mathbf{M}_{-\mathrm{y}}$	$\mathbf{M}_{+\mathrm{x}}$	$\mathbf{M}_{-\mathrm{x}}$
χ_{xxx}	χ_{xxx}	$-\chi_{xxx}$	0	0
χ_{xyy}	χ_{xyy}	$-\chi_{xyy}$	0	0
χ_{xzz}	χ_{xzz}	$-\chi_{xzz}$	0	0
χ_{xyz}	0	0	0	0
χ_{xxz}	χ_{xxz}	χ_{xxz}	χ_{yyz}	χ_{yyz}
χ_{xxy}	0	0	$-\chi_{yxy}$	χ_{yxy}
χ_{yxx}	0	0	$-\chi_{xyy}$	χ_{xyy}
χ_{yyy}	0	0	$-\chi_{xxx}$	χ_{xxx}
χ_{yzz}	0	0	$-\chi_{xzz}$	χ_{xzz}
χ_{yyz}	χ_{yyz}	χ_{yyz}	χ_{xxz}	χ_{xxz}
χ_{yxz}	0	0	0	0
χ_{yxy}	χ_{yxy}	$-\chi_{yxy}$	0	0
χ_{zxx}	χ_{zxx}	χ_{zxx}	χ_{zyy}	χ_{zyy}
χ_{zyy}	χ_{zyy}	χ_{zyy}	χ_{zxx}	χ_{zxx}
χ_{zzz}	χ_{zzz}	χ_{zzz}	χ_{zzz}	χ_{zzz}
χ_{zyz}	0	0	$-\chi_{zxz}$	χ_{zxz}
χ_{zxz}	χ_{zxz}	$-\chi_{zxz}$	0	0
χ_{zxy}	0	0	0	0

Supplementary Table 3. Allowed SHG tensor components in the four magnetic states of CeAlSi and their symmetry relationship.

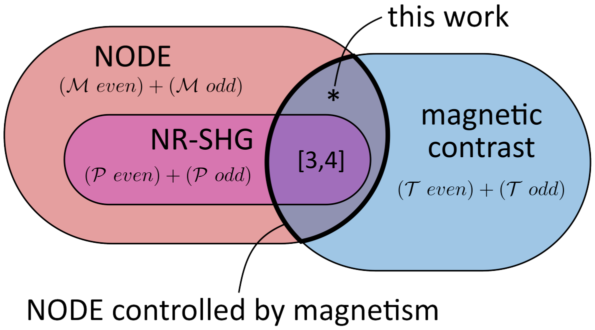

Supplementary Fig. 3. **SHG interference phenomena** Interference between different contributions to the total SHG response can give rise to a variety of optical phenomena. The phenomena can be distinguished based on the microscopic origin of the contributions. Magnetic contrast arises from a mixing of contributions that are even/odd under time reversal $\mathcal{T}$; nonreciprocal SHG arises from mixing SHG contributions that are even/odd under spatial inversion $\mathcal{P}$; the NODE emerges from interference between SHG contributions that are even/odd under a mirror operation $\mathcal{M}$. Previous demonstrations of NR-SHG are in the cross section of all three phenomena [3, 4]. As a broken inversion symmetry (required for $\mathcal{P}$ odd SHG) implies a broken mirror symmetry, any material that exhibits NR-SHG also allows for a NODE, i.e. NODE is a generalization of NR-SHG.

between i-type (time reversal $\mathcal{T}$ even) and c-type ($\mathcal{T}$ odd) SHG contributions. Below the magnetic ordering temperature T_{C}, the nonlinear optical susceptibility can be expressed as

$$\chi_{ijk}(T<T_{\mathrm{C}}) = \underbrace{\chi_{ijk}(T>T_{\mathrm{C}})}_{\text{crystallographic (i−type) SHG}} + \underbrace{\chi_{ijkl}(T>T_{\mathrm{C}})M_l,}_{\text{magnetic (c−type) SHG}} \tag{6}$$

where the indices i,j,k,l refer to the crystallographic directions x, y, or z. Since the measured SHG intensity is proportional to $|\chi_{ijk}(T<T_{\mathrm{C}})|^2$, we find that the SHG intensity depends on the interference between crystallographic and magnetic SHG contributions and therefore on the direction of $\boldsymbol{M}$. This gives rise to the observed domain contrast. The domain contrast, in turn, depends on the relative amplitude between the crystallographic and magnetic SHG tensor contributions. The contrast is maximized if crystallographic and magnetic SHG have the same amplitude.

2. **Nonreciprocal SHG** can occur if the source term $\boldsymbol{S}$ in the electromagnetic wave equation contains contributions that are both even and odd functions of the wave vector $\boldsymbol{k}$, such that

$$\boldsymbol{S} = \underbrace{\boldsymbol{S}_{\mathrm{even}}}_{\text{k−even (electric dipole) SHG}} + \underbrace{\boldsymbol{S}_{\mathrm{odd}}}_{\text{k−odd (magnetic dipole) SHG}} . \tag{7}$$

Nonreciprocal SHG is not possible to observe in the electric-dipole approximation as all electric-dipole contributions are $\boldsymbol{k}$-even. Often, k-even and k-odd contributions correspond to electric dipole and magnetic dipole SHG, respectively [3, 4], but also higher order multipole contributions can exist [5]. The precise structure of $\boldsymbol{S}_{\mathrm{even}}$ and $\boldsymbol{S}_{\mathrm{odd}}$ depends on the involved multipole contribution. In terms of point group symmetry operations, k-odd contributions like magnetic-dipole SHG are allowed in centrosymmetric materials and are hence $\mathcal{P}$ even. Moreover, the presence of k-even SHG contributions requires a broken inversion symmetry. They are thus $\mathcal{P}$ odd. Whereas the magnetic domain contrast arises from interference between $\mathcal{T}$ even and $\mathcal{T}$ odd SHG contributions, the observation of NR-SHG requires interference between $\mathcal{P}$ even and $\mathcal{P}$ odd SHG contributions, which highlights the different microscopic origin.

3. We introduce the **nonlinear optical diode effect (NODE)** as a third SHG interference phenomenon. We will provide here both a phenomenological as well as a microscopic definition. Phenomenologically, we define the NODE as an effect whereby a nonlinear optical response (such as SHG), is stronger for one light propagation direction than for the reversed light propagation direction. Such an effect can be defined both in reflection and in transmission geometry (Supp. Fig. 4). In both geometries, we can define a directional contrast η as

$$\eta = (I^{\rightarrow} - I^{\leftarrow}) \,/\, (I^{\rightarrow} + I^{\leftarrow})\,. \tag{8}$$

Microscopically, the reversal of the propagation direction is related to a mirror operation $\mathcal{M}$ (the relevant mirror plane runs vertically in Supp. Fig. 4a and b). The NODE arises if the total SHG response is comprised of contributions that are even and odd under that mirror operation $\mathcal{M}$.

$$\chi_{ijk} = \chi_{ijk}(\mathcal{M}\ even) + \chi_{ijk}(\mathcal{M}\ odd) \tag{9}$$

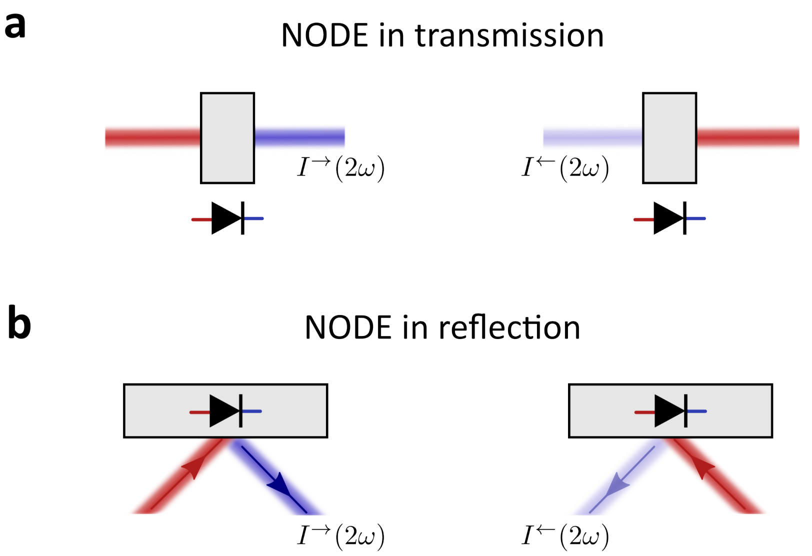

Supplementary Fig. 4. **Definition of the NODE.** We define the **nonlinear optical diode effect (NODE)** purely phenomenologically as an effect whereby a nonlinear optical response (such as SHG), is stronger for one light propagation direction than for the reversed light propagation direction. Such an effect can be defined both in **a,** transmission and in **b,** reflection geometry. In both cases, we can define a directional contrast η.

While nonreciprocal SHG is crucial for the occurrence of a NODE in transmission, the minimal example in Supplementary Fig. 5 illustrates that a NODE can be observed in reflection even in the electric dipole approximation. This shows that the NODE and nonreciprocal SHG are independent effects that rely on the transformation behavior of the SHG response under different operations. In this example, the relevant mirror operation that enables the NODE is $\mathcal{M}_x$. The component χ_{xxx} is odd under $\mathcal{M}_x$, whereas χ_{xxz} is even. Note that the NODE is present in Supplementary Fig. 5a,b even if χ_{xxx} and χ_{xxz} are i-type. Thus, the NODE is also distinct from the magnetic domain contrast as a NODE does not strictly require breaking of time-reversal symmetry or c-type SHG contributions [6]. The presence of a magnetic domain contrast also does not imply the presence of a NODE [7, 8] (both c-type and i-type SHG contributions may have the same transformation behavior with respect to $\mathcal{M}$). Hence, the magnetic contrast in Supp. Fig. 3 is overlapping, but not contained in the NODE. In the cross section, the magnetic orders allows to control the directionality of the NODE, as demonstrated in the present work.

To summarize, magnetic contrast, NR-SHG, and NODE are distinct interference phenomena that rely on different microscopic symmetries. Magnetic contrast strictly requires a broken time-reversal symmetry $\mathcal{T}$ and NR-SHG can only be observed in materials with broken spatial inversion $\mathcal{P}$. The NODE requires neither symmetry to be broken, but is related to mirror symmetries. Note that $\mathcal{P} = \mathcal{M}_x \circ \mathcal{M}_y \circ \mathcal{M}_z$. Thus, a broken spatial inversion symmetry (as required for NR-SHG) implies a broken mirror symmetry, which enables a NODE. Therefore, NR-SHG is fully contained in the NODE in Supp. Fig. 3.

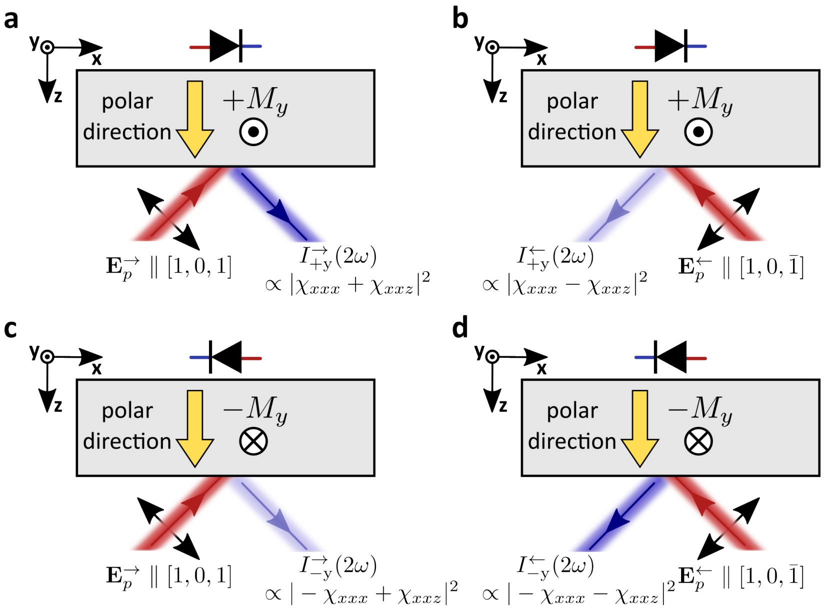

Supplementary Fig. 5. **Minimal example for the observation and manipulation of the NODE.** We consider a fictitious noncentrosymmetric material for which χ_{xxx} and χ_{xxz} are the only nonvanishing SHG tensor components in an orientation analogous to Fig. 1d. For p-polarized light, the electric field $\mathbf{E}_p(\omega)$ is in general parallel to $\mathbf{k} \times \hat{\mathbf{y}}$. The detected SHG intensity is proportional to the induced nonlinear polarization $I(2\omega) \propto |\sum_i P_i(2\omega)|^2 \propto |\sum_{i,j,k} \chi_{ijk} E_j(\omega) E_k(\omega)|^2 = |\chi_{xxx} E_{p,x}^2 + \chi_{xxz} E_{p,x} E_{p,z}|^2$. Specifically for an angle of incidence of 45°, the electric field is parallel to **a,** $[1,0,1]$ in forward direction and **b,** parallel to $[1,0,\bar{1}]$ in backward direction. Thus, $I^{\rightarrow}(2\omega) \propto |\chi_{xxx} + \chi_{xxz}|^2$ and $I^{\leftarrow}(2\omega) \propto |\chi_{xxx} - \chi_{xxz}|^2 \neq I^{\rightarrow}(2\omega)|^2$. We therefore observe a NODE in this minimal example. **c,d,** In a material where χ_{xxz} arises from a polar crystal structure whereas χ_{xxx} is due to magnetic order (as in the case of CeAlSi), switching the magnetization reverses the directionality of the NODE and thus allows its manipulation.

2. POLARIZATION-RESOLVED SHG MEASUREMENTS

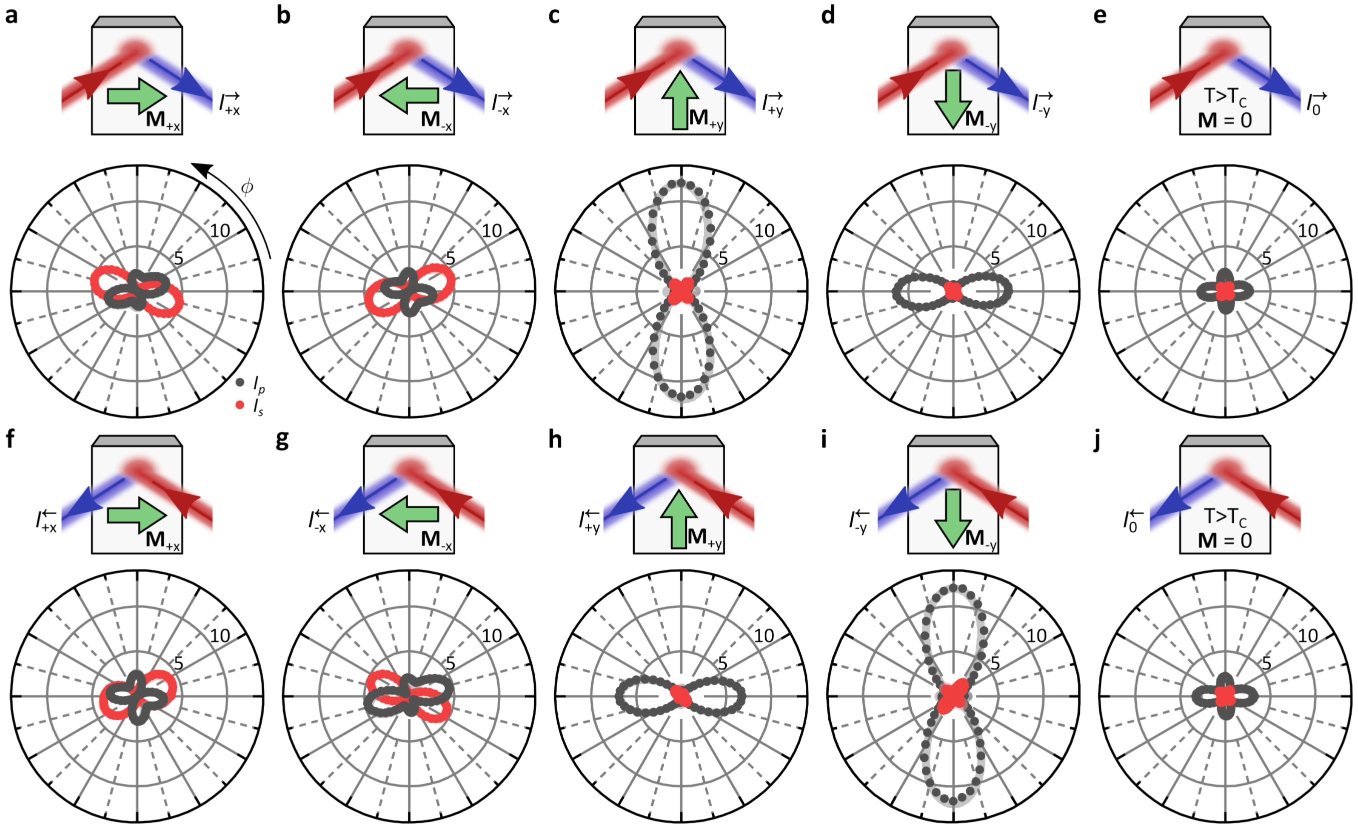

Supplementary Fig. 6. **SHG Polarization dependencies. a-e,** in forward direction and **f-j,** in backward direction. $I^{\mathbf{k}}_{\mathbf{M}}(\phi) = I^{-\mathbf{k}}_{-\mathbf{M}}(\phi)$ always holds (see Supplementary Fig. 15 for details). Moreover, reversing the light propagation direction is equivalent to reversing the magnetization. All measurements were taken at $\hbar\omega = 715\,\text{meV}$.

We show in Supplementary Fig. 6 the polarization dependence of the SHG intensity for the different magnetic states at $3\,\text{K} < T_\text{C}$ and in the paramagnetic phase at $12\,\text{K} > T_\text{C}$ ($T_\text{C} = 8.2\,\text{K}$, see below). We show the polarization dependence for both forward (Fig. 6a-e) and backward (Fig. 6f-j) propagating light.

The polarization dependencies were measured such that the incident polarization was rotated, while the outgoing p (grey) and s (red) polarized SHG was detected. All measurements were taken at $\hbar\omega = 715\,\text{meV}$.

In all magnetic states, we observe a NODE for some light polarization. In the paramagnetic state, we observe an absence of a NODE for all polarizations. Moreover, we note certain symmetries between the polarization dependencies. In particular, $I^{\mathbf{k}}_{\mathbf{M}}(\phi) = I^{-\mathbf{k}}_{-\mathbf{M}}(\phi)$ always holds (see Supplementary Fig. 15 for details).

All 16 measurements in the magnetic phase ('4 magnetic states' x '2 SHG polarizations' x '2 propagation directions') were fit simultaneously with one consistent set of SHG tensor components for ED-SHG according to point group $2'mm'$ (Supplementary Table 3). To accommodate experimental uncertainties, we allow for a small rotation of the sample about the x, y, and z axis corresponding to a pitch of the sample, a deviation of the angle of incidence from 45°, and an in-plane rotation of the sample, respectively. All deviations from the ideal case are below 2°. The fit results are shown as solid lines in Supplementary Fig. 6. Note that the fit curves are often overlapping with the data points.

χ_{ijk}	$\lvert\chi_{ijk}\rvert/\lvert\chi_{zzz}\rvert$	$(\Phi_{ijk} - \Phi_{zzz})/\pi$
zzz	1	0
xxz	0.2	-0.58
zxx	0.29	0.28
yyz	0.76	-0.56
zyy	1.87	0.76
xxx	0.06	0.64
yyx	0.62	-0.55
xyy	1.99	0.77
zzx	0.78	0.55
xzz	2.36	-0.17

Supplementary Table 4. Magnitudes and phases of χ tensor components relative to χ_{zzz}. Values were found by fitting all 168 measurements below T_C in Supplementary Fig. 6 simultaneously with one consistent set of χ tensor components according to point group $2'mm'$.

The excellent quality of the fit indicates that the detected SHG is dominated by ED-SHG. We provide in table 4 the magnitudes and phases for the fitted tensor $\chi_{ijk} = |\chi_{ijk}| * e^{i\Phi_{ijk}}$ components relative to χ_{zzz}.

Based on the obtained relative magnitudes and phases of all tensor components, we can calculate the SHG intensity and the directional contrast even in configurations that are not easily experimentally accessible. For example, we show in Supplementary Fig. 7 the incident angle dependence of the SHG intensity for s-polarized incident light and p-polarized outgoing SHG (experimental configuration of Fig. 1e in the main text). Although we observe a strong directional contrast at an incident angle of 45°, this angle is in general not optimal. As the directional contrast must be a continuous function of the incident angle, it is interesting to consider two edge case:

1. <u>Normal incidence (AoI = 0°)</u>: In this scenario, incident and outgoing beams overlap. A reversal of the light path (AoI $\rightarrow$ -AoI) does not change the experiment. Hence, a NODE cannot be observed in normal incidence reflection.

2. <u>Grazing incidence (AoI = 90°))</u>: Also here, a NODE cannot be observed in the electric-dipole approximation. In the coordinate system of Supplementary Fig. 5, grazing incidence corresponds to light propagation parallel to $\hat{\mathbf{x}}$. The transversal electric fields thus only couple to tensor components χ_{ijk} with $i, j, k \in \{y, z\}$, which are all even with respect to the mirror operation $\mathcal{M}_x$. Hence, the basic symmetry requirement for the observation of a NODE (Eq. (9)) cannot be realized.

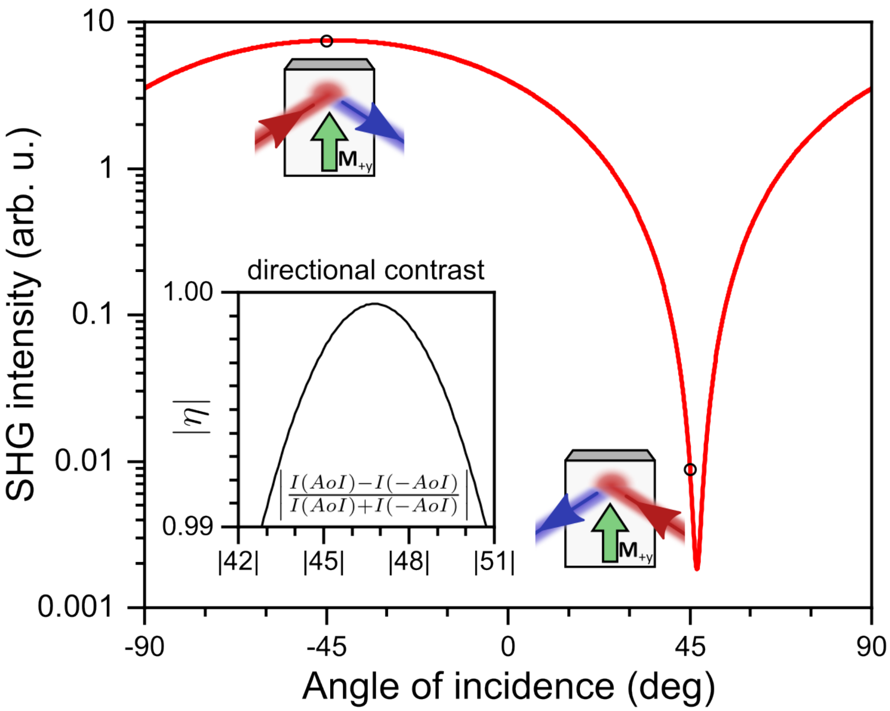

Supplementary Fig. 7. **Incident angle dependence.** Calculated incident angle dependence based on the relative magnitudes and phases of the SHG tensor component extracted from experimental polarization dependencies. An angle of incidence of 45° is not optimal. By adjusting the angle of incidence, a numerically determined directional contrast > 99.9% could be possible (inset). Black circles mark the experimental configuration of ±45°.

3. TEMPERATURE DEPENDENT CHARACTERIZATION

Unless explicitly noted differently, all measurements were performed at a sample temperature of 3 K. We provide here additional details on the temperature-dependent characterization of CeAlSi.

We show in the main text that we can switch the magnetization of CeAlSi and control the NODE by applying current (Fig. 3 in the main text). In Supplementary Fig. 8a, we show the measured SHG intensity at various temperatures during the application of a DC current. Here, an external magnetic field of 30 mT stabilizes a ferromagnetic $\mathbf{M}_{+y}$ state such that no current-induced switching can occur. We can clearly distinguish the paramagnetic phase (homogeneously light blue) from the ferromagnetically ordered phase (white to red).

Note that ”Temperature” here refers to the temperature set point instead of the actual sample temperature, which is elevated due to current-induced ohmic heating. The effect of ohmic heating can be seen from the approximately parabolic current-induced temperature increase. This increase is further independent of the applied current direction (consistent with ohmic heating). Under maximum current, the sample enters the paramagnetic phase at a temperature of about 4 K. Thus. at 3 K, the sample remains in the magnetically ordered phase for all applied DC currents.

Note also that the SHG intensity in the paramagnetic phase is independent of the applied current. In contrast, the SHG intensity at maximum current in Fig. 3c in the main text is different for the opposite polarities. Thus, the sample remained in the magnetically ordered state during the measurement of Fig. 3c.

Supplementary Fig. 8b shows the temperature dependence of the SHG intensity in the absence of applied cur-

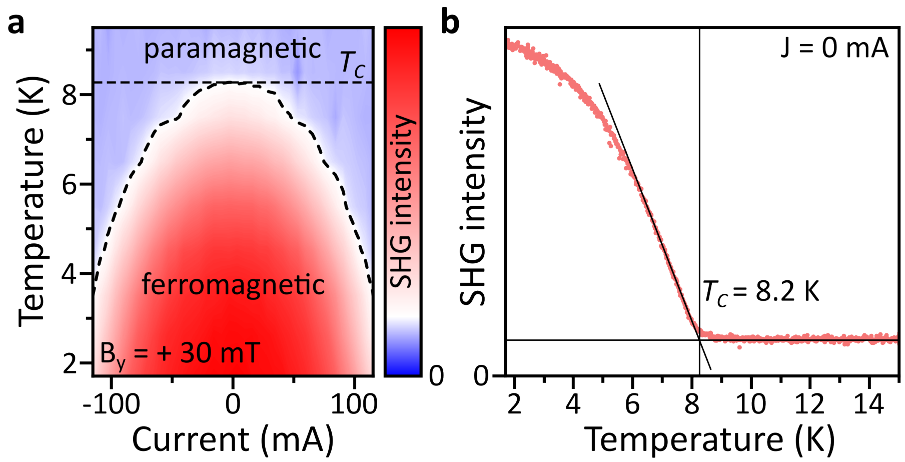

Supplementary Fig. 8. **Temperature dependent characterization.** **a,** SHG intensity as a function of temperature and applied electric DC current. For temperatures below 4 K, the sample remains in the ferromagnetically ordered phase for all applied currents. **b,** Temperature dependent SHG intensity in the absence of electric current. We find a magnetic transition temperature of $T_C = 8.2\,\mathrm{K}$ in agreement with literature [2].

rent. We find a transition temperature of 8.2 K in agreement with previous characterizations [2, 9]. As CeAlSi is noncentrosymmetric above T_C, we continue to observe a finite SHG intensity above 8.2 K.

4. ADDITIONAL NODE SPECTROSCOPY

We report in the main text the observation of a nonlinear optical diode effect (NODE) over a wide spectral range. We demonstrate this observation in the main text on the basis of selected measurements in certain magnetic configurations. In this section, we provide additional systematic experimental evidence in complementary settings. The striking agreement between symmetry-related measurements is evidence for a high data quality and shows that the observations are intrinsic properties of CeAlSi.

A. Observation of a broadband NODE in the $\mathbf{M}_{-\mathrm{y}}$ state

We show in Supplementary Fig. 9 SHG spectra for counter-propagating light beams. The sample is in a magnetic $\mathbf{M}_{-\mathrm{y}}$ state. Complementary to Fig. 1e of the main text, we again observe a broadband NODE. However, the spectra are inverted relative to Fig. 1e of the main text, which were recorded in the $\mathbf{M}_{+\mathrm{y}}$ state. This observation is immediate evidence that the magnetization in CeAlSi controls the directionality of the broadband NODE.

Other experimental parameters are identical: spectra were recorded at 3 K, incident fundamental light is s polarized, and we detect outgoing p polarized SHG light.

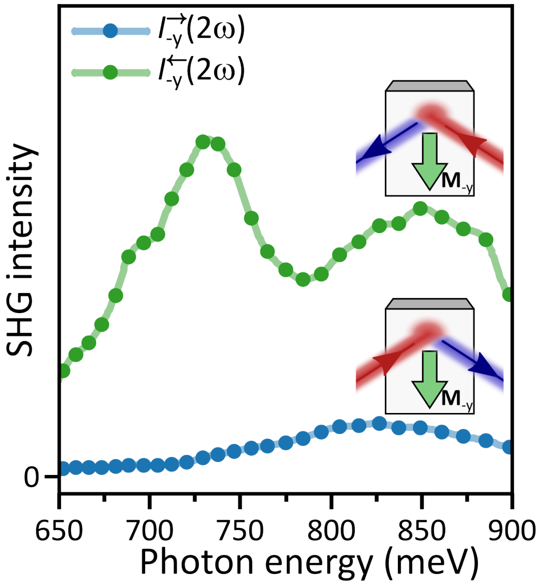

Supplementary Fig. 9. **Observation of a broadband NODE in the** $\mathbf{M}_{-\mathrm{y}}$ **state.** $I^{\rightarrow}_{-\mathrm{y}}(2\omega) \ll I^{\leftarrow}_{-\mathrm{y}}(2\omega)$ over a broad range $> 250\,\mathrm{meV}$.

B. Determination and robustness of directional contrast

We introduce in the main text the directional contrast

$$\eta = \frac{I_{+y}^{\rightarrow} - I_{+y}^{\leftarrow}}{I_{+y}^{\rightarrow} + I_{+y}^{\leftarrow}}. \tag{10}$$

By symmetry, this expression is equivalent to

$$\eta = \frac{I_{+y}^{\rightarrow} - I_{-y}^{\rightarrow}}{I_{+y}^{\rightarrow} + I_{-y}^{\rightarrow}} \tag{11}$$

$$= -\frac{I_{+y}^{\leftarrow} - I_{+y}^{\leftarrow}}{I_{+y}^{\leftarrow} + I_{+y}^{\leftarrow}} \tag{12}$$

$$= -\frac{I_{-y}^{\rightarrow} - I_{-y}^{\leftarrow}}{I_{-y}^{\rightarrow} + I_{-y}^{\leftarrow}} \tag{13}$$

$$\tag{14}$$

Here, we experimentally verify this equivalence. We show in Supplementary Fig. 10a and b 2D maps of the detected p polarized SHG intensity as a function of incident photon energy and polarization angle (0°/180° - p polarized; 90° - s polarized fundamental light). Both maps were obtained with light propagating in forward direction, however, the sample is in a magnetic $\mathbf{M}_{+\mathrm{y}}$ state in Supplementary Fig. 10a and in a $\mathbf{M}_{-\mathrm{y}}$ state in Supplementary Fig. 10b. We notice clear differences between the two panels.

Supplementary Fig. 10c shows the directional contrast according to Eq. 11 showing a strong contrast (for certain polarizations) at all photon energies. To verify the robustness of the directional contrast, i.e., the equivalence of Eqs. 10-13, we evaluated the SHG intensity also in the reversed direction for both magnetic states (Supplementary Fig. 10d and e).

We notice the (expected) similarity between Supplementary Fig. 10a and e, as well as Supplementary Fig. 10b and d. Based on these four measurements, we can determine the directional contrast η according to all four definitions in Eqs. 10-13. Indeed, as we can see from Supplementary Figs. 10c, f, g, and h, the obtained values for η agree well (up to a sign). The absolute value of Supplementary Fig. 10c is shown in Fig. 4a in the main text.

In Supplementary Fig. 10i, we display for all photon energies within the considered spectral range the maximum contrast that is achievable by selecting a specific incident polarization. We note that the maximum of $|\eta|$ determined according to Eqs. 11 and 12 show a striking agreement, whereas the values determined according to Eqs. 10 and 13 differ slightly. This is due to experimental uncertainties in reversing the beam path, which we can circumvent by determining η from measurements in the same propagation direction but opposite magnetic states. Note that this options exists in CeAlSi as reversing the propagation direction is equivalent to reversing the beam path (see Sec. 7).

C. Directional contrast in the $\mathbf{M}_{\pm\mathrm{x}}$ states

The discussion of the directional contrast in the main text and in the previous section was centered on the magnetic $\mathbf{M}_{\pm\mathrm{y}}$ states. In this section, we present an analogous analysis for the $\mathbf{M}_{\pm\mathrm{x}}$ states.

In Supplementary Fig. 11a-d, we show 2D maps of the SHG intensity as a function of incident fundamental photon energy and polarization. The panels show data recorded in forward direction in the $\mathbf{M}_{+\mathrm{x}}$ state (panel a), in forward direction in the $\mathbf{M}_{-\mathrm{x}}$ state (panel b), in reversed direction in the $\mathbf{M}_{+\mathrm{x}}$ state (panel c), and in reversed direction in

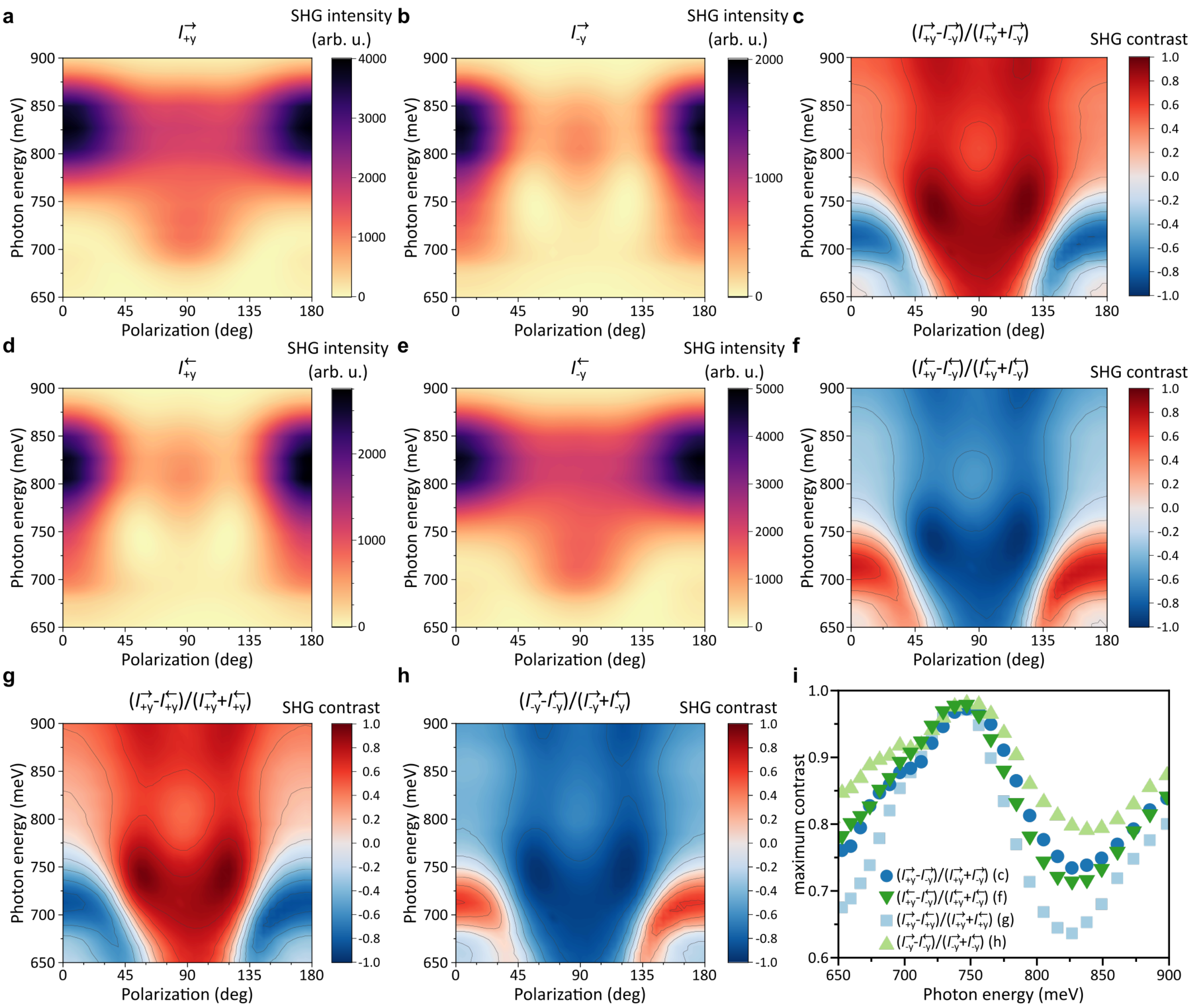

Supplementary Fig. 10. **Determination and robustness of directional contrast (Fig. 4a). a,** 2D map of measured p polarized SHG intensity in forward direction as a function of incident polarization and photon energy for the $\mathbf{M}_{+\mathrm{y}}$ state and **b,** $\mathbf{M}_{-\mathrm{y}}$ state. **c,** directional contrast η as defined by Eq. 11. **d-f,** analogous to **a-c** in backward direction. **g,h,** directional contrast defined as contrast between opposite directions in the same magnetic state. Panels c, f, g, and h are all consistent, demonstrating the robustness of determining the directional contrast. **i,** Maximum contrast analogous to Fig. 4b for panels **c**, **f**, **g**, and **h**. Maximum contrast extracted from **c**, **f** agree well, whereas experimental uncertainties in reversing the beam path cause slight deviations between **g** and **h**. The absolute value of panel **c** is shown in Fig. 4a.

the $\mathbf{M}_{-\mathrm{x}}$ state (panel d). We recognize the clear (expected) pairwise similarity between panels a and d, as well as panels b and c.

As we showed in the previous section that a determination of η according to Eqs. 10-13 yields analogous results, we restrict ourselves here to Eq. 11. The obtained 2D map for the directional contrast is shown in Supplementary Fig. 11e. By selecting specific incident light polarizations, we can ensure a directional contrast of at least 72% for all

photon energies and up to 96.6%, very similar to the achievable contrast in the magnetic $\mathbf{M}_{\pm y}$ states (73% to 97.2%).

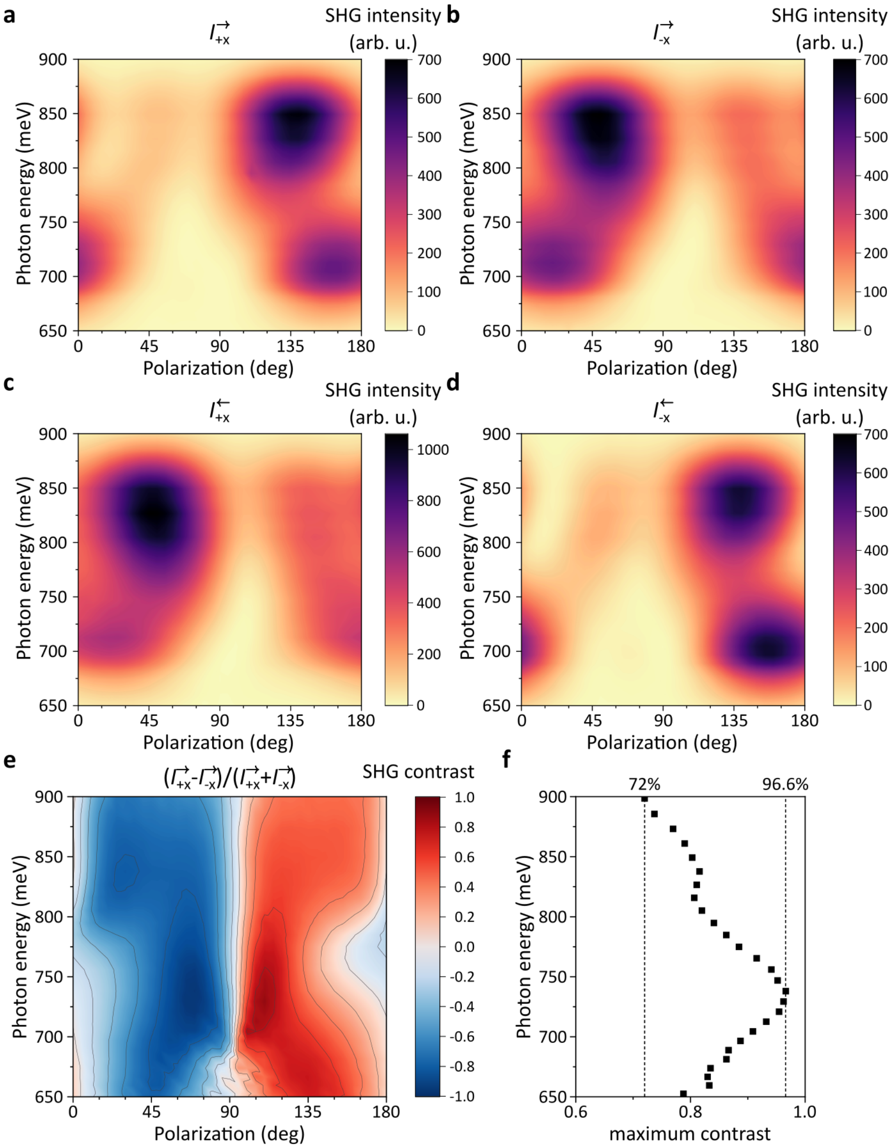

Supplementary Fig. 11. **Directional contrast in the $\mathbf{M}_{\pm x}$ states. a,** 2D map of measured s polarized SHG intensity as a function of incident polarization and photon energy in forward direction for the $\mathbf{M}_{+x}$ state and **b,** $\mathbf{M}_{-x}$ state and in reversed direction for **c,** the $\mathbf{M}_{+x}$ state and **d,** the $\mathbf{M}_{-x}$ state. **e,** directional contrast η as defined by Eq. 11. **f,** Maximum achievable contrast analogous to Fig. 4b obtain from panel e.

D. A note on symmetry

By comparing the 2D maps of the directional contrast in the $\mathbf{M}_{\pm x}$ states (Supplementary Fig. 11e) and $\mathbf{M}_{\pm y}$ states (e.g, Supplementary Fig. 10c), we recognize differences in the symmetry of these magnetic states. In the $\mathbf{M}_{\pm y}$ states, the 2D map is mirror symmetric about the 0° and 90° polarization directions. This is due to the mirror symmetry of the polarization dependence in the $\mathbf{M}_{\pm y}$ states (Supplementary Fig. 6), which is microscopically a consequence of

the m_y mirror symmetry in these states. In contrast, in the $\mathbf{M}_{\pm y}$ states, the 2D map is mirror symmetric with a sign change, which is an experimental manifestation of the m'_y symmetry in these states ($m'_y = m_y \circ \mathcal{T}$).

E. Directional contrast in the paramagnetic phase

Here, we verify that the NODE vanishes in the paramagnetic phase of CeAlSi as required by symmetry (Sec. 7) and indicated by the polarization-dependent measurements at 715 meV (Supplementary Fig. 6e,f).

In Supplementary Fig. 12, we show 2D maps showing the detected SHG intensity as a function of incident polarization and photon energy. All measurements were taken at 12 K, which is well above $T_C = 8.2$ K (Sec. 3). We consider here both p and s polarized SHG light for both forward and backward propagating light. For both polarizations, measurements with forward and backward propagating light are strikingly similar. We note that for the s polarized SHG response (Supplementary Fig. 12b,d), the overall SHG intensity is relatively low. Therefore, experimental uncertainties in reversing the light become apparent in the measurement. Moreover, in particular for the 0° and 90° polarization, the SHG intensity approaches zero (as required by 4mm symmetry), which impedes a meaningful determination of the directional contrast. We therefore refrain from showing 2D maps for η, but note the both qualitative and quantitative similarity between forward and backward propagating SHG responses for both s and p polarized SHG light.

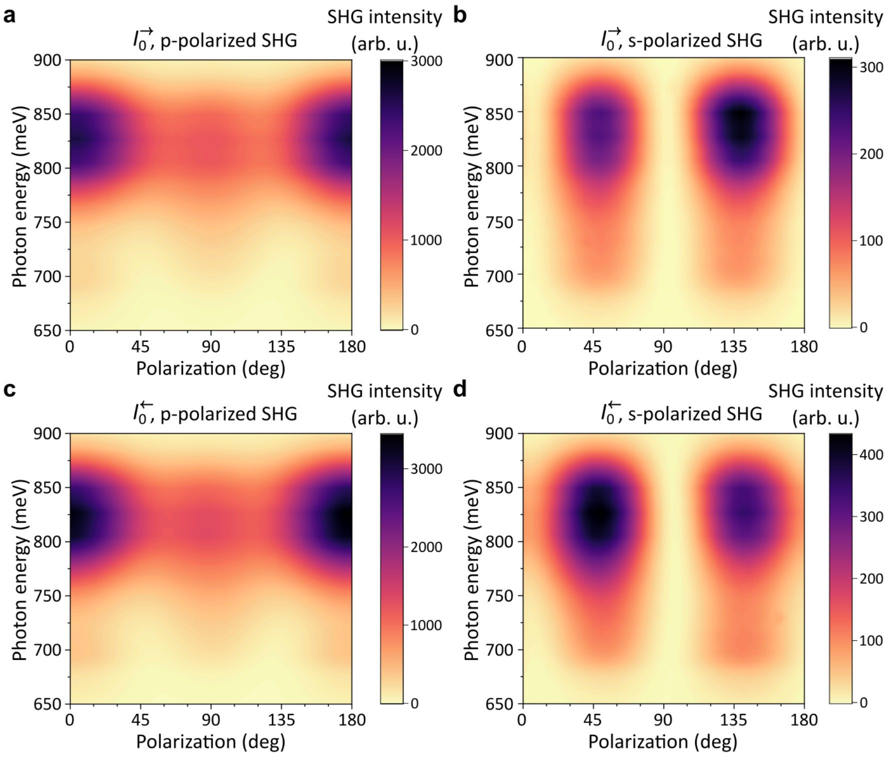

Supplementary Fig. 12. **Vanishing NODE in the paramagnetic phase.** SHG intensity as a function of incident light polarization and photon energy for **a,** p polarized SHG in forward direction **b,** s polarized SHG in forward direction **c,** p polarized SHG in reversed direction and **d,** s polarized SHG in reversed direction. Comparing opposite propagation directions for each SHG polarization, we not the absence of a discernible NODE.

5. DOMAIN ASSIGNMENT AND MAPPING

For a fixed propagation direction, we can utilize the SHG domain contrast in CeAlSi to visualize the four different magnetic states. In particular, the unique polarization dependence of the SHG in all four magnetic states (Supplementary Fig. 6) allows us to identify four distinct combinations of incident fundamental and outgoing SHG polarizations that are uniquely sensitive to each of the magnetic states (Supplementary Fig. 13). This enables the capability to image the magnetic domain distribution of CeAlSi and assign the magnetic states.

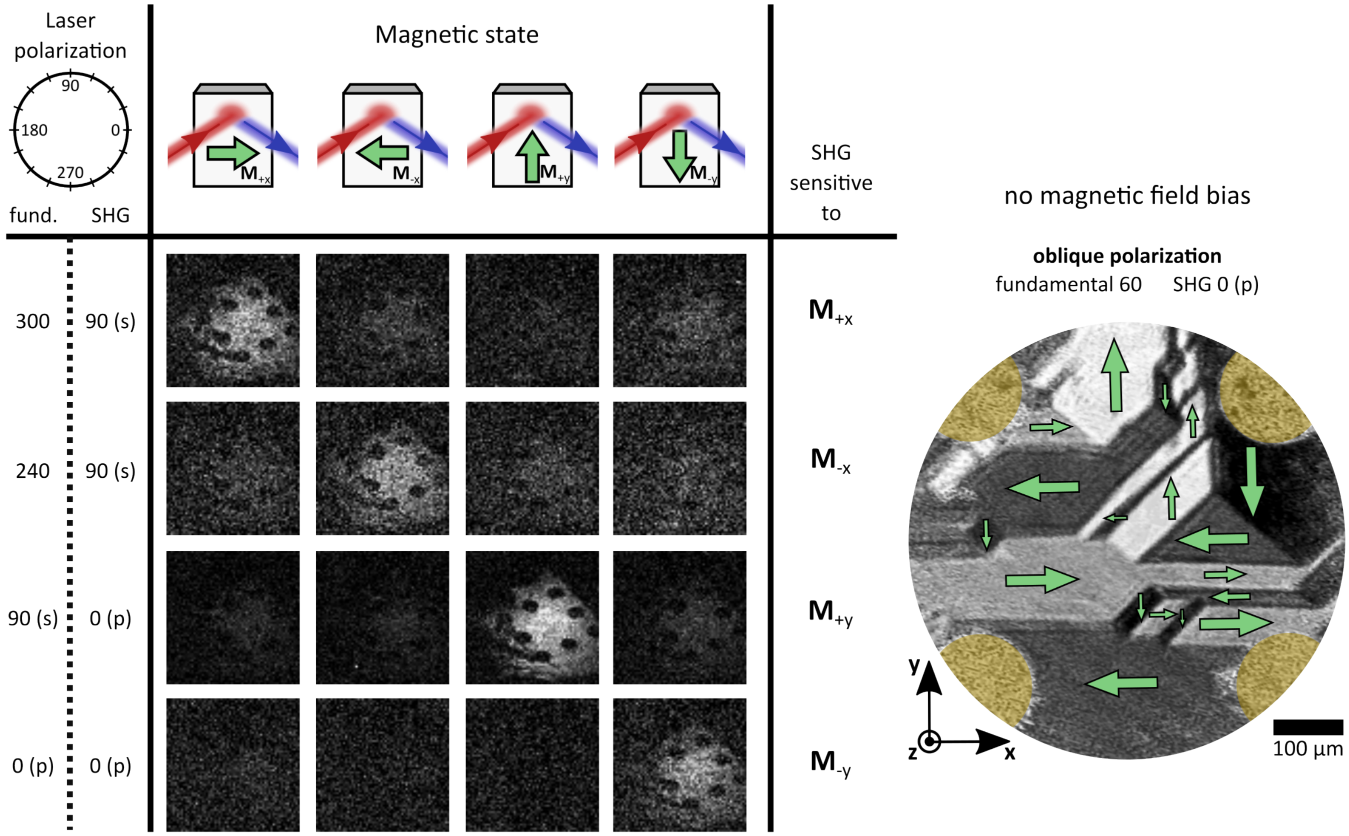

Supplementary Fig. 13. **Domain assignment.** The unique polarization dependence of the SHG in all four magnetic states (Supplementary Fig. 6) allows us to identify four distinct combinations of incident fundamental and outgoing SHG polarizations that are uniquely sensitive to each of the magnetic states. Here, an external magnetic field of approximately 30 mT ensures a well-defined magnetic single-domain state. We were thus able to verify that the current-induced switching in Fig. 3c is between $\mathbf{M}_{\pm\mathrm{y}}$ states. In the absence of a magnetic field bias, we can identify an oblique polarization angle, where the intensity of all four magnetic states is different and assign the domains (right hand side). Yellow circles show positions of gold electrodes.

We frequently exploit the capability to map and assign domain states in CeAlSi. For example:

1. It enables the *operando* visualization of the domain distribution during the application of current (Supplementary Movie 3).
2. It allows us to verify that the current-induced switching in Fig. 3c of the main text is between $\mathbf{M}_{\pm\mathrm{y}}$ states.
3. It allows us to verify that the current-induced switching in Fig. 3g of the main text is between $\mathbf{M}_{\pm\mathrm{x}}$ states.

4. It enables the visualization of magnetic-field induced switching (Section 6)

To obtain the domain image on the right-hand side of Supplementary Fig. 13, we normalized the SHG image recorded at 3 K to the SHG image recorded at 12 K. Thus, spatial inhomogeneities in the SHG intensity due to the approximately Gaussian beam profile are removed. Since such a normalization is only possible within the illuminated area, the image is cropped to area approximately corresponding to the beam spot.

6. MAGNETIC FIELD DEPENDENT SHG CHARACTERIZATION

Utilizing the capability to selectively visualize magnetic domains by SHG imaging (Sec. 5), we determined the domain state for magnetic fields in the range of $\pm 20\,\mathrm{mT}$. The measurement was performed at 1.6 K. We show the area fraction of the different states in Supplementary Fig. 14a.

We chose here two setting for the laser polarization: (1) the 90/0 configuration that yields a high SHG intensity only for $\mathbf{M}_{+\mathrm{y}}$ domains and (2) the 90/90 configuration yields a high SHG intensity for both $\mathbf{M}_{+\mathrm{x}}$ and $\mathbf{M}_{-\mathrm{x}}$ domains. The area fraction for $\mathbf{M}_{-\mathrm{y}}$ domains was obtained numerically by completing the determined area fractions to 1.

Consistent with literature, we find a narrow hysteresis from which we deduce a low coercive field of 1-2 mT (estimated from the hysteresis at an area fraction of 50%). Note that the $\mathbf{M}_{+\mathrm{y}}$ hysteresis loop is not symmetric around 0 mT due to CeAlSi exhibiting four magnetic states. Thus, we expect an area fraction of 25% for all domains in a fully randomized state at 0 mT (i.e., 25% $\mathbf{M}_{+\mathrm{y}}$, 25% $\mathbf{M}_{-\mathrm{y}}$, and 50% $\mathbf{M}_{+\mathrm{x}} + \mathbf{M}_{-\mathrm{x}}$). Thus, the $\mathbf{M}_{+\mathrm{y}}$ ($\mathbf{M}_{-\mathrm{y}}$) hysteresis loop appears shifted towards positive (negative) magnetic fields. Moreover, we find that $\mathbf{M}_{\pm\mathrm{x}}$ domains are only present at low magnetic fields and fully suppressed for $|B_y| > 10\,\mathrm{mT}$.

In panels b-e of Supplementary Fig. 14, we show SHG domain images at selected magnetic fields for laser polarization setting (1) sensitive only to $\mathbf{M}_{+\mathrm{y}}$ domains. At $-20\,\mathrm{mT}$, the sample appears homogenously dark (panel b). In contrast, at 20 mT, the sample appears homogenously bright (panel d). These states correspond to single domain $\mathbf{M}_{+\mathrm{y}}$ and $\mathbf{M}_{-\mathrm{y}}$ states, respectively. Panels c and e show the domain image at 2.7 mT taken while increasing and decreasing the magnetic field, respectively. As expected, the area fraction of $\mathbf{M}_{+\mathrm{y}}$ domains is larger after decreasing the field from 20 mT (panel e). The series of all images for both laser polarization settings are combined into Supplementary Movies 1 (polarization setting 1) and 2 (polarization setting 2).

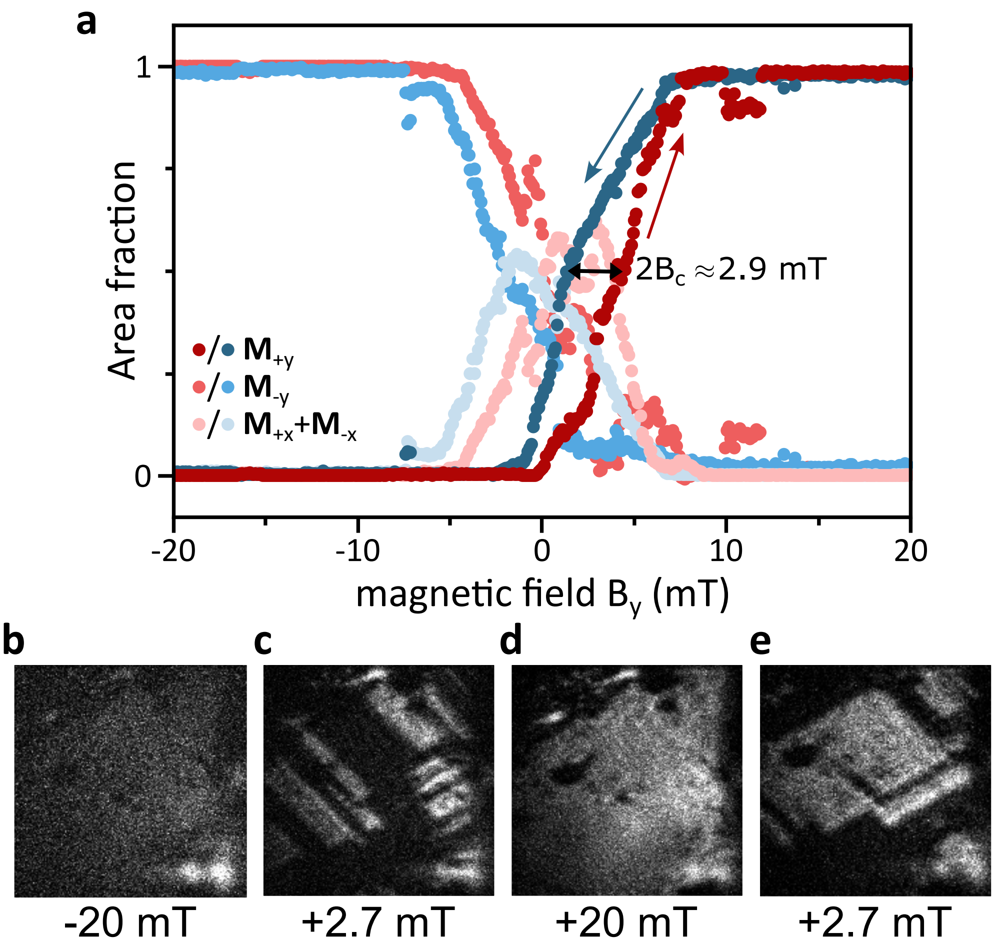

Supplementary Fig. 14. **Magnetic field dependent characterization. a,** magnetic hysteresis. Red shaded data points are taken with increasing field; blue shaded data points with decreasing field. Consistent with literature [2], we find that CeAlSi is a soft magnet with a coercive field of just a few mT. **b-e,** magnetic domain imaging by SHG. The laser polarization is chosen such that bright areas correspond $\mathbf{M}_{+y}$ domains. The area fractions in panel a were extracted from such images.

7. SYMMETRY OF THE NODE

In addition to the discussions in the main text, we discuss here the symmetry of the NODE in more detail. We find that mirror symmetries of the paramagnetic crystal lattice restore certain symmetries in the SHG polarization dependencies (Fig. 2 in the main text and Supplementary Fig. 6). To see this, we consider the effect of the mirror operations m_x and m_y on the magnetization, propagation direction, and light polarization (Supplementary Fig. 15).

In general, the mirror operation m_x reverses the propagation direction ($\mathbf{k} \rightarrow -\mathbf{k}$) (Supplementary Fig. 15a and c) while the mirror operation m_y preserves $\mathbf{k}$ (Supplementary Fig. 15a and c). Both mirror operations individually transform the linear light polarization from an angle ϕ to $-\phi$ relative to p-polarized light. We will consider in the following discussion the SHG intensity $I^{\mathbf{k}}_{\mathbf{M}}(\phi)$. The arguments below are valid both for s and p-polarized SHG light.

In the $\mathbf{M}_{\pm x}$ states (Supplementary Fig. 15a), the mirror operation m_x preserves the magnetization along $\hat{\mathbf{x}}$ ($\mathbf{M}_{\pm x} \rightarrow \mathbf{M}_{\pm x}$). Therefore, the SHG intensity $I^{\mathbf{k}}_{\mathbf{M}}(\phi)$ has the symmetry $I^{\mathbf{k}}_{\mathbf{M}}(\phi) = I^{-\mathbf{k}}_{\mathbf{M}}(-\phi)$. As a consequence in the experiment, the polarization dependencies in Supplementary Figs. 6a and b are mirror symmetric to Supplementary Figs. 6f and g.

In contrast to m_x, m_y reverses the magnetization along $\hat{\mathbf{x}}$ as shown in Supplementary Fig. 15b ($\mathbf{M}_{\pm x} \rightarrow \mathbf{M}_{\mp x}$). Thus, $I^{\mathbf{k}}_{\mathbf{M}}(\phi) = I^{\mathbf{k}}_{-\mathbf{M}}(-\phi)$. Therefore, Supplementary Figs. 6a and f are mirror symmetric to Supplementary Figs. 6b and g.

The situation in the $\mathbf{M}_{\pm y}$ states is slightly different. As shown in Supplementary Fig. 15c, m_x reverses the magnetization along $\hat{\mathbf{y}}$ ($\mathbf{M}_{\pm y} \rightarrow \mathbf{M}_{\mp y}$). Thus, $I^{\mathbf{k}}_{\mathbf{M}}(\phi) = I^{-\mathbf{k}}_{-\mathbf{M}}(-\phi)$ (Supplementary Figs. 6c and d are symmetric to of Supplementary Figs. 6i and h.

Finally, m_y preserves the magnetization along $\hat{\mathbf{y}}$ as illustrated in Supplementary Fig. 15d ($\mathbf{M}_{\pm y} \rightarrow \mathbf{M}_{\pm y}$). Thus, $I^{\mathbf{k}}_{\mathbf{M}}(\phi) = I^{\mathbf{k}}_{\mathbf{M}}(-\phi)$. Therefore, Supplementary Figs. 6(c,d,h,i) are individually mirror symmetric.

Irrespective of the magnetic state, the application of both mirror operations (equivalent to a two-fold rotational axis 2_z along the $\hat{\mathbf{z}}$ axis) in any magnetic state always enforces $I^{\mathbf{k}}_{\mathbf{M}}(\phi) = I^{-\mathbf{k}}_{-\mathbf{M}}(\phi)$. Thus, reversing both the magnetization and the propagation direction always preserves the SHG intensity for any polarization dependence. Note that this property hinges on the presence of a two-fold rotational axis 2_z along the $\hat{\mathbf{z}}$ axis in the paramagnetic phase. For a general material, it is therefore not automatically guaranteed that reversing the magnetization is equivalent to reversing the light propagation. As a further consequence of the 2_z axis in the paramagnetic phase, the NODE is absent in the paramagnetic phase.

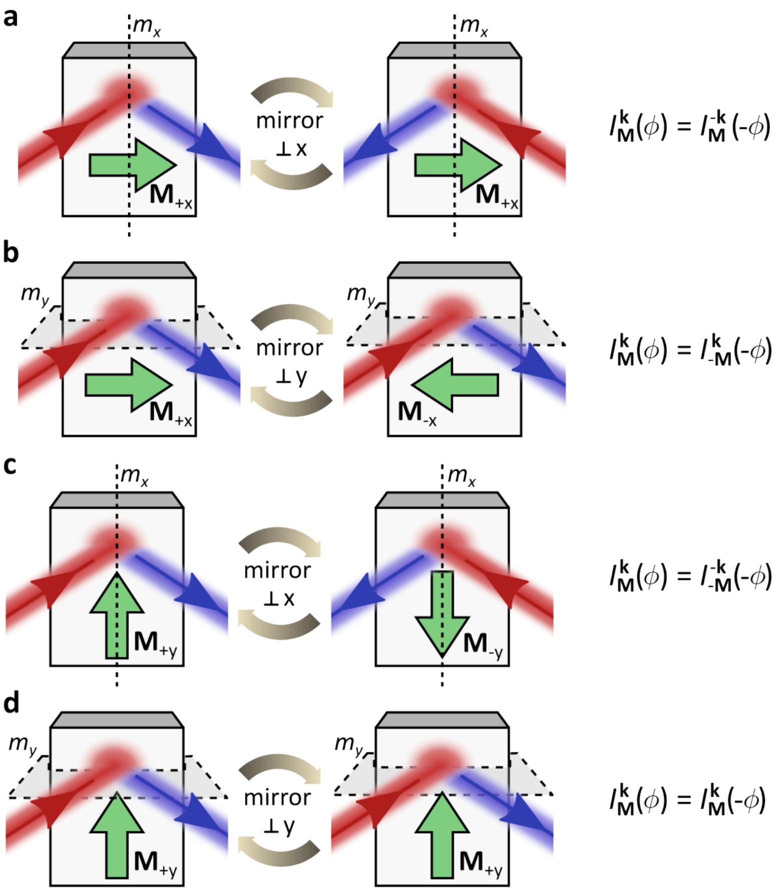

Supplementary Fig. 15. **Consequences of mirror symmetries in CeAlSi. a,** In the $\mathbf{M}_{\pm x}$ states, m_x preserves the magnetization along $\hat{\mathbf{x}}$ ($\mathbf{M}_{\pm x} \to \mathbf{M}_{\pm x}$). Therefore, the SHG intensity $I^{\mathbf{k}}_{\mathbf{M}}(\phi)$ has the symmetry $I^{\mathbf{k}}_{\mathbf{M}}(\phi) = I^{-\mathbf{k}}_{\mathbf{M}}(-\phi)$ (Supplementary Figs. 6(a,b) are mirror symmetric to Supplementary. 6(f,g)). **b,** m_y reverses the magnetization along $\hat{\mathbf{x}}$ ($\mathbf{M}_{\pm x} \to \mathbf{M}_{\mp x}$). Thus, $I^{\mathbf{k}}_{\mathbf{M}}(\phi) = I^{\mathbf{k}}_{-\mathbf{M}}(-\phi)$ (Supplementary Figs. 6(a,f) are mirror symmetric to Supplementary Figs. 6(b,g)). **c,** In the $\mathbf{M}_{\pm y}$ states, m_x reverses the magnetization along $\hat{\mathbf{y}}$ ($\mathbf{M}_{\pm y} \to \mathbf{M}_{\mp y}$).Thus, $I^{\mathbf{k}}_{\mathbf{M}}(\phi) = I^{-\mathbf{k}}_{-\mathbf{M}}(-\phi)$ (Supplementary Figs. 6(c,d) are symmetric to of Supplementary Figs. 6(i,h)). **d,** m_y preserves the magnetization along $\hat{\mathbf{y}}$ ($\mathbf{M}_{\pm y} \to \mathbf{M}_{\pm y}$). Thus, $I^{\mathbf{k}}_{\mathbf{M}}(\phi) = I^{\mathbf{k}}_{\mathbf{M}}(-\phi)$ (Supplementary Figs. 6(c,d,h,i) are individually mirror symmetric). The application of both mirror operations in any magnetic state enforces $I^{\mathbf{k}}_{\mathbf{M}}(\phi) = I^{-\mathbf{k}}_{-\mathbf{M}}(\phi)$. Thus, reversing both the magnetization and the propagation direction always preserves the SHG polarization dependence.

8. INTEGRATED SHG SPECTRUM

In analogy to the NDD as a polarization-independent, directional contrast in the absorption coefficient, we introduced in the main text the integrated SHG intensity as a polarization-independent nonlinear optical observable. In this section, we present additional data illustrating properties of the integrated SHG intensity. In particular, we show that the NODE is present even for the integrated SHG intensity and it is thus not restricted to specific incident and outgoing light polarizations.

We define the integrated SHG intensity as the intensity measured without polarization analysis (thus detecting simultaneously s and p polarized SHG light) and averaged over all incident polarization angles ϕ. The such defined observable is independent of both incoming and outgoing light polarizations.

As we showed in Sec. 7 and Supplementary Fig. 6, the detected SHG intensity in the $\mathbf{M}_{\pm\mathrm{x}}$ states exhibits the symmetry $I^{\mathbf{k}}_{\pm\mathrm{x}}(\phi) = I^{-\mathbf{k}}_{\pm\mathrm{x}}(-\phi) = I^{\mathbf{k}}_{\mp\mathrm{x}}(-\phi)$. Averaging over all polarization angles ϕ, we find $\langle I^{\mathbf{k}}_{\pm\mathrm{x}}\rangle = \langle I^{-\mathbf{k}}_{\pm\mathrm{x}}\rangle = \langle I^{\mathbf{k}}_{\mp\mathrm{x}}\rangle$, i.e., the integrated SHG intensity in the $\mathbf{M}_{\pm\mathrm{x}}$ states is independent of the propagation direction and magnetization direction. Accordingly, we find in Supplementary Fig. 16a equal integrated SHG intensities in the $\mathbf{M}_{\pm\mathrm{x}}$ states.

In contrast, the integrated SHG intensity in the $\mathbf{M}_{\pm\mathrm{y}}$ states is different. This is due to the absence of a symmetry operation that relates the two magnetic states while preserving the propagation direction. We have either $I^{\mathbf{k}}_{\pm\mathrm{y}}(\phi) = I^{-\mathbf{k}}_{\mp\mathrm{y}}(-\phi)$ or $I^{\mathbf{k}}_{\pm\mathrm{y}}(\phi) = I^{\mathbf{k}}_{\pm\mathrm{y}}(-\phi)$ (Sec. 7). Thus, for a fixed propagation direction, the integrated SHG intensity can differ for the two magnetic states $\mathbf{M}_{+\mathrm{y}}$ and $\mathbf{M}_{-\mathrm{y}}$.

Reversing the light propagation (Supplementary Fig. 16b), we find in agreement with the symmetry considerations equal integrated SHG intensities for the $\mathbf{M}_{\pm\mathrm{x}}$ states, but the relation between $\langle I_{+\mathrm{y}}\rangle$ and $\langle I_{-\mathrm{y}}\rangle$ is inverted relative to panel a. This property is a direct consequence of the relation $I^{\mathbf{k}}_{\pm\mathrm{y}}(\phi) = I^{-\mathbf{k}}_{\mp\mathrm{y}}(-\phi)$.

In Supplementary Fig. 16c, we show the spectral evolution of the integrated SHG intensity for one propagation direction. Very strikingly, we find $\langle I^{\rightarrow}_{+x}\rangle = \langle I^{\rightarrow}_{-x}\rangle$ for all photon energies.

Overall, we note the following properties of the integrated SHG intensity:

- if the SHG intensity does not exhibit a NODE (here: SHG response above T_C), the integrated SHG intensity does not exhibit a NODE.
- if the SHG intensity shows a NODE, mirror symmetries may still enforce a vanishing NODE in the integrated SHG intensity.

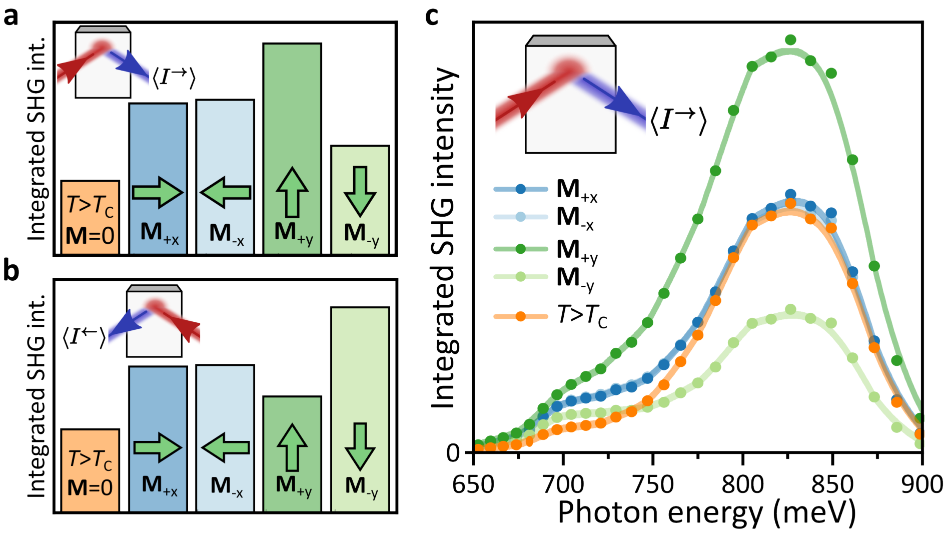

Supplementary Fig. 16. **NODE in the integrated SHG intensity. a,** Integrated SHG in forward direction at 715 meV. **b,** Integrated SHG in backward direction at 715 meV. In contrast to the forward direction, $\langle I^{\leftarrow}_{+y}\rangle < \langle I^{\leftarrow}_{-y}\rangle$. The integrated SHG intensity in the $\mathbf{M}_{\pm x}$ states is always identical for the two propagation directions. **c,** Spectra of the integrated SHG intensity $\langle I^{\rightarrow}\rangle$ in forward direction for all magnetic states and in the paramagnetic phase. $\langle I^{\rightarrow}_{+x}\rangle = \langle I^{\rightarrow}_{-x}\rangle$ due to m_y mirror operation (Sec. 7).

9. FIB SAMPLE

In an effort to demonstrate the NODE in a device-like structure and minimize the effect of Joule heating observed in the current switching of the bulk single crystal, we microstructured a CeAlSi crystal into a free-standing bar of approximately 126 µm length, 29 µm width, and 2 µm thickness by focused-ion-beam (FIB) milling (see methods for details). A false-colored SEM picture is shown in Fig. 3f in the main text. In order to identify the magnetic state during the application of electrical current (Fig. 3g in the main text), we pre-characterized the SHG response of the FIB device by applying a static magnetic field of approximately 30 mT along the four magnetic easy axes. The incident polarization dependence for the s and p polarized SHG light is shown in Supplementary Fig. 17.

Although the polarization dependencies differ from the scans on the bulk single crystal (Supplementary Fig. 6), we can identify four significantly different polarization patterns for the four magnetic states. Possible reasons for the differences between Supplementary Figs. 6 and 17 include changes to the surface morphology of the material caused by FIB milling (see methods) and deviations of the angle of incidence from 45° (a few degree tilt between the surface of the substrate and the surface of the FIB device was unavoidable due to the extreme aspect ratio of the FIB sample).

A comparison of the magnetic-field poled polarization dependencies in Supplementary Fig. 17 and the electric current induced states (Fig. 3h,i in the main text) reveals a current-induced switching between $\mathbf{M}_{+\mathrm{x}}$ and $\mathbf{M}_{-\mathrm{x}}$. Solid lines in Supplementary Fig. 17 are fits. In analogy to Supplementary Fig. 6, we fit all measurements simultaneously with one consistent set of χ tensor components. We list the fit parameters relative to χ_{zzz} in Supplementary Table 5 below. A surface tilt of approximately 8° was included in the fitting procedure. The excellent fit quality is consistent with the $2'mm'$ symmetry for the magnetic state of the FIB sample.

Moreover, we performed a temperature-dependent characterization of the FIB sample. A magnetic field of approximately 30 mT stabilizes a magnetic $\mathbf{M}_{+\mathrm{y}}$ state. In Supplementary Fig. 18a, we show the SHG intensity as a function of temperature in the absence of any electrical current. We find a transition temperature in agreement with the bulk sample.

In Supplementary Fig. 18b, we show the SHG intensity in the $\mathbf{M}_{+\mathrm{y}}$ state at 3 K under the application of an electric DC current. We extrapolate a critical current of 8.5 mA above which Joule heating raises the sample temperature above the transition temperature. Joule heating is negligible for the currents relevant for Fig. 3g in the main text ($< 3\,\mathrm{mA}$).

χ_{ijk}	$\lvert\chi_{ijk}\rvert/\lvert\chi_{zzz}\rvert$	$(\Phi_{ijk} - \Phi_{zzz})/\pi$
zzz	1	0
xxz	0.069	0.774
zxx	0.313	0.182
yyz	0.106	-0.268
zyy	0.284	0.377
xxx	0.081	-0.26
yyx	0.05	-0.11
xyy	0.204	0.366
zzx	0.125	0.605
xzz	0.127	-0.08

Supplementary Table 5. Magnitudes and phases of χ tensor components relative to χ_{zzz}. Values were found by fitting all 8 measurements below T_C in Supplementary Fig. 17 simultaneously with one consistent set of χ tensor components according to point group $2'mm'$.

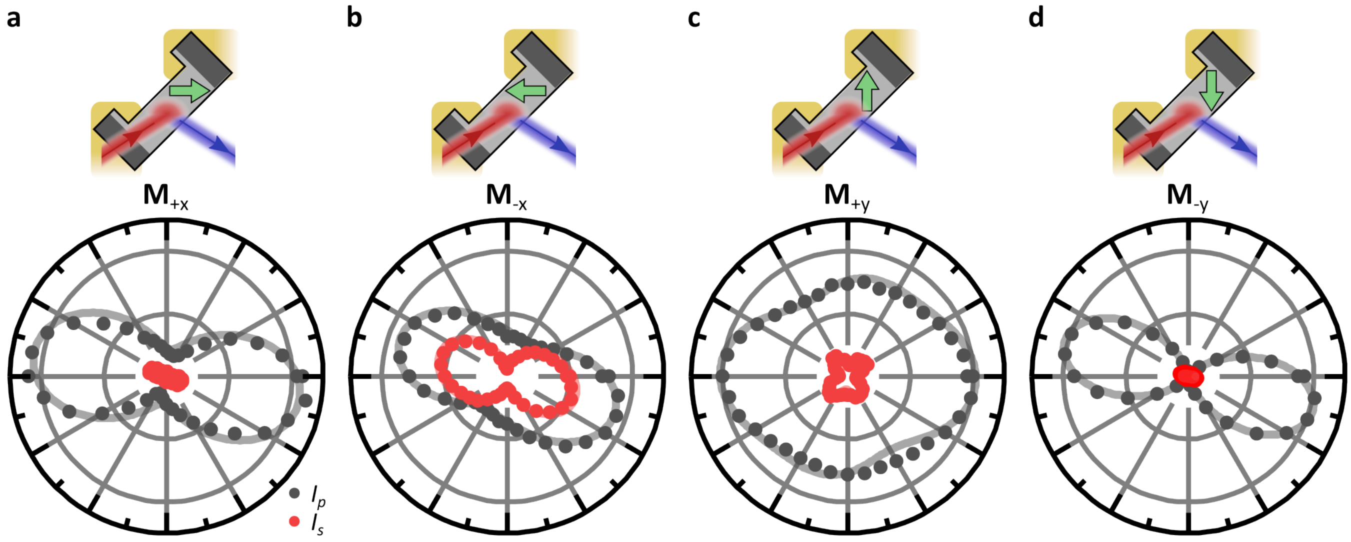

Supplementary Fig. 17. **SHG polarization dependencies for FIB sample. a-d** SHG polarization dependence in the magnetic $\mathbf{M}_{+\mathrm{x}}$, $\mathbf{M}_{-\mathrm{x}}$, $\mathbf{M}_{+\mathrm{y}}$, and $\mathbf{M}_{-\mathrm{y}}$ states, respectively. Analogously to the SHG signal from CeAlSi bulk single crystals (Supplementary Fig. 6), the SHG polarization dependence allows us to distinguish the four magnetization directions in micro-machined CeAlSi samples. Here, the magnetic state was stabilized by an external magnetic field of 30 mT. Solid lines are fits. A comparison of field-polarized measurements to the field-free measurements shown in Figs. 3h,i reveals electrical switching between $\mathbf{M}_{-\mathrm{x}}$ and $\mathbf{M}_{+\mathrm{x}}$ states, respectively.

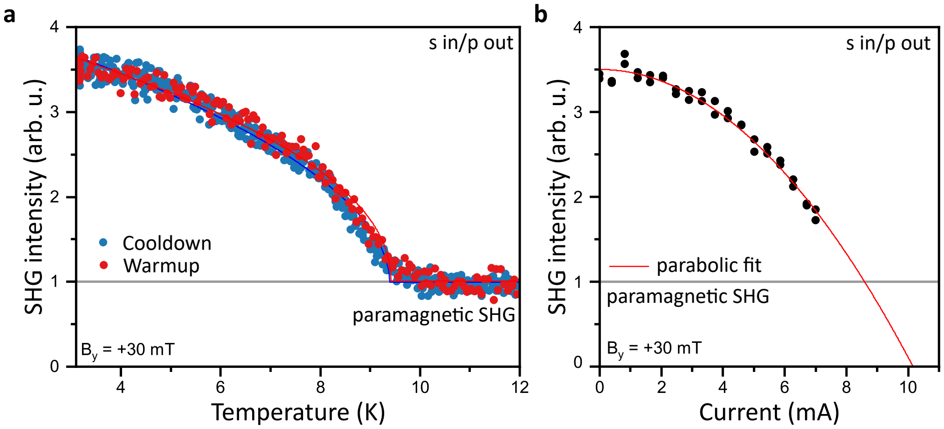

Supplementary Fig. 18. **Temperature-dependent characterization of the FIB sample.** In analogy to the SHG response of CeAlSi bulk single crystals (Supplementary Fig. 8), we performed **a,** a temperature-dependent characterization of the FIB sample and **b,** characterized the effect of Joule heating on the FIB sample. We find a transition temperature in agreement with the bulk. From panel b, we find that the FIB sample remains in the magnetically ordered phase below 8.5 mA. The effect of Joule heating is negligible below 3 mA. The magnetic state was stabilized by an external magnetic field of 30 mT.

10. FIRST-PRINCIPLES CALCULATIONS

A. Electronic structure of CeAlSi

We provide here further details about the first-principles calculations described in the main text. In particular, in this section we describe the electronic structure of CeAlSi in more detail based on numerical first-principles calculations. We performed calculations both in the paramagnetic and in the ferromagnetically ordered phase of CeAlSi. A comparison of the band structure is shown in Supplementary Fig. 19. Our calculations agree well with other recent calculations and experimental observations [10].

Due to the noncentrosymmetric lattice structure, CeAlSi is already a Weyl semimetal in the paramagnetic phase. Going to the ferromagnetic phase, CeAlSi remains to be a Weyl semimetal, with relatively small modifications to the details of the band structure. Figure 19 shows that the overall band structures of the paramagnetic and ferromagnetic phases are similar: CeAlSi is a semimetal in both states.

We found a total of 40 Weyl nodes in the band structure of CeAlSi in the paramagnetic phase. We list their positions and energies in Table 6 and illustrate their positions in Supplementary Fig. 20. The magnetic order does not change the number of Weyl nodes, but the position of the 40 Weyl nodes shifts both in energy and in momentum upon the inclusion of the magnetic order (Table 7).

Based on the electronic structure in the magnetically ordered phase, we calculate the components of the nonlinear optical susceptibility χ_{ijk} following the diagrammatic approach to nonlinear optical responses [11, 12] (see also methods). We show the real and imaginary parts of the obtained susceptibilities in supplementary Fig. 21 in the energy range of 650 meV to 900 meV (incident photon energy). We find that only the components that are allowed by symmetry exhibit finite susceptibilities [1].

We note here also the absence of any sharp resonances in any tensor component. This is in contrast to wide bandgap insulators, where often sharp resonances occur in the energy range close to the band gap [13]. Our numerical calculations thus reproduce a broadband SHG response. In Supplementary Fig. 22, we show the calculated spectrum of the SHG intensity in a setting that reproduces the experiment in Fig. 1e of the main text (s polarized incident light, p polarized SHG response, 45° angle of incidence, $\mathbf{M}_{+\mathrm{y}}$ state). We observe a clear difference in the SHG intensity between the two propagation directions. As a consequence of the absence of resonances in χ_{ijk}, the SHG intensity evolves smoothly as a function of photon energy.

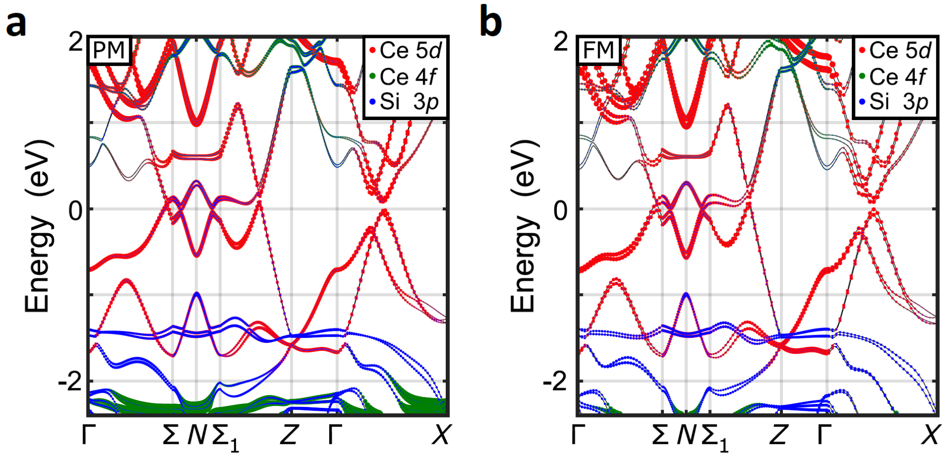

Supplementary Fig. 19. Band structure of CeAlSi in the **a,** paramagnetic phase and **b,** ferromagnetic phase. Color coded is the orbital composition of the electronic bands.

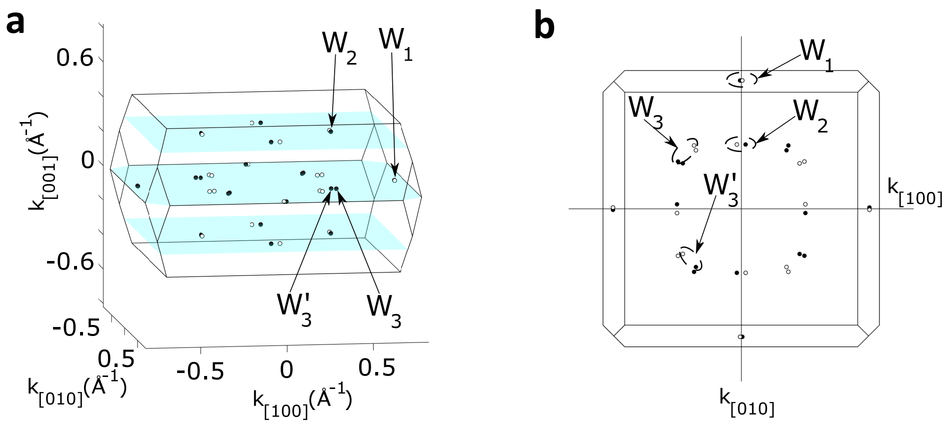

Supplementary Fig. 20. **Distribution of Weyl points in the Brillouin zone. a,** The distribution of the 40 Weyl nodes in the Brillouin zone. Black and white colors represent opposite Weyl node chirality. **b,** 2D projection of all Weyl nodes.

Weyl Nodes	Position: $(k_x, k_y, k_z)(1/\text{Å})$		Energy (meV)
	Chirality: -1	Chirality: +1	
W_1	$(0.007, 0.744, 0.000)$	$(-0.744, -0.007, 0.000)$	58
	$(0.744, -0.007, 0.000)$	$(0.007, -0.744, 0.000)$	58
	$(-0.007, -0.744, 0.000)$	$(0.744, 0.007, 0.000)$	58
	$(-0.744, 0.007, 0.000)$	$(-0.007, 0.744, 0.000)$	58
W_2	$(-0.026, 0.372, \pm0.295)$	$(-0.372, 0.026, \pm0.295)$	31
	$(0.372, 0.026, \pm0.295)$	$(-0.026, -0.372, \pm0.295)$	31
	$(0.026, -0.372, \pm0.295)$	$(0.372, -0.026, \pm0.295)$	31
	$(-0.372, -0.026, \pm0.295)$	$(0.026, 0.372, \pm0.295)$	31
W_3	$(0.274, -0.367, 0.000)$	$(0.367, -0.274, 0.000)$	46
	$(-0.367, -0.274, 0.000)$	$(0.274, 0.367, 0.000)$	46
	$(0.367, 0.274, 0.000)$	$(-0.274, -0.367, 0.000)$	46
	$(-0.274, 0.367, 0.000)$	$(-0.367, 0.274, 0.000)$	46
$W_3^{'}$	$(0.339, 0.263, 0.000)$	$(-0.263, -0.339, 0.000)$	31
	$(-0.339, -0.263, 0.000)$	$(0.263, 0.339, 0.000)$	31
	$(0.263, -0.339, 0.000)$	$(0.339, -0.263, 0.000)$	31
	$(-0.263, 0.339, 0.000)$	$(-0.339, 0.263, 0.000)$	31

Supplementary Table 6. **Weyl node positions and energies in the paramagnetic phase of CeAlSi.** Weyl node energies are given relative to the Fermi energy.

Weyl Nodes	Position: $(k_x, k_y, k_z)(1/\text{Å})$		Energy(meV)
	Chirality: -1	Chirality: +1	
W_1	$(0.005, 0.760, 0.000)$	$(-0.760, -0.005, 0.000)$	39
	$(0.757, -0.004, 0.000)$	$(0.004, -0.757, 0.000)$	65
	$(-0.007, -0.738, 0.000)$	$(0.738, 0.007, 0.000)$	70
	$(-0.743, 0.004, 0.000)$	$(-0.004, 0.743, 0.000)$	81
W_2	$(-0.023, 0.395, \pm0.284)$	$(-0.395, 0.023, \pm0.284)$	-24
	$(0.388, 0.023, \pm0.282)$	$(-0.023, -0.388, \pm0.282)$	-23
	$(0.018, -0.369, \pm0.305)$	$(0.369, -0.018, \pm0.305)$	23
	$(-0.372, -0.024, \pm0.294)$	$(0.024, 0.372, \pm0.294)$	26
W_3	$(0.276, -0.366, 0.000)$	$(0.366, -0.276, 0.000)$	8
	$(-0.355, -0.282, 0.000)$	$(0.282, 0.355, 0.000)$	37
	$(0.387, 0.267, 0.000)$	$(-0.267, -0.387, 0.000)$	48
	$(-0.270, 0.372, 0.000)$	$(-0.372, 0.270, 0.000)$	69
$W_3^{'}$	$(0.333, 0.257, 0.000)$	$(-0.257, -0.333, 0.000)$	10
	$(-0.340, -0.257, 0.000)$	$(0.257, 0.340, 0.000)$	26
	$(0.270, -0.339, 0.000)$	$(0.339, -0.270, 0.000)$	33
	$(-0.278, 0.353, 0.000)$	$(-0.353, 0.278, 0.000)$	52

Supplementary Table 7. **Weyl node positions and energies in the ferromagnetic phase of CeAlSi.** Weyl node energies are given relative to the Fermi energy.

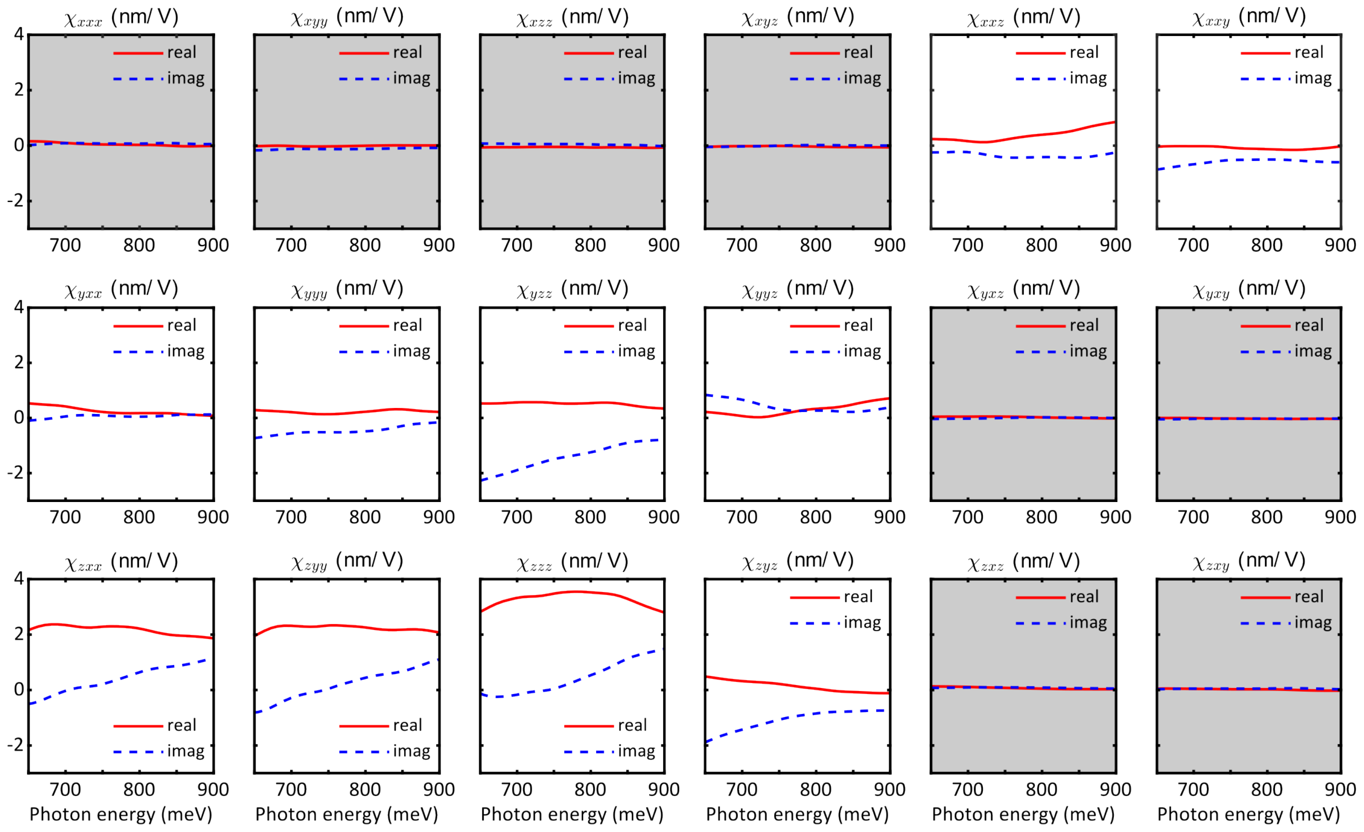

Supplementary Fig. 21. **First-principles calculations of SHG tensor component spectra in the magnetic phase of CeAlSi.** All spectra are shown on the same scale. In agreement with the $2'mm'$ point group symmetry, the grayed out components vanish. The allowed components evolve continuously without any pronounced resonance in agreement with our understanding of broadband SHG due to linearly dispersive bands in CeAlSi.

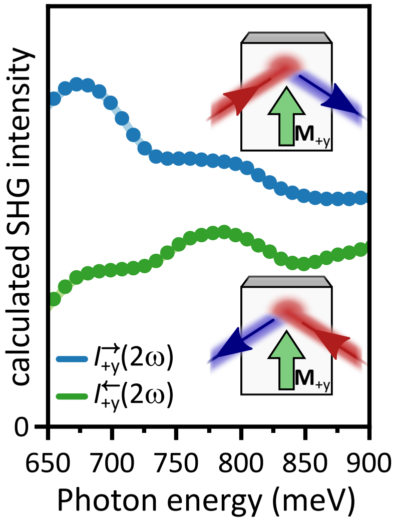

Supplementary Fig. 22. **Observation of the NODE from first principles.** Spectral dependence of SHG intensity for counter-propagating light paths. The calculations are done based on the numerically obtained tensor components (Supplementary Fig. 21) and reproducing the experimental setting of Fig. 1e in the main text. We observe a clear NODE.

B. k-space resolved χ contributions

As described in the methods section, we calculate χ as $\chi_{ijk} = \int_{BZ} d^3\mathbf{k}\xi_{ijk}$, where $\xi_{ijk} = \xi^I_{ijk} + \xi^{II}_{ijk}$ with

$$\xi^I_{ijk} = C \sum_{m \neq n} f_{mn} \left(\frac{h^{ij}_{nm} h^k_{mn} + h^{ik}_{nm} h^j_{mn}}{\omega + i\eta - \omega_{mn}} + \frac{h^{jk}_{mn} h^i_{nm}}{2\omega + i\eta - \omega_{mn}} \right), \tag{15}$$

$$\xi^{II}_{ijk} = C \sum_{m \neq n \neq p} \frac{h^i_{pm} \left(h^j_{mn} h^k_{np} + h^k_{mn} h^j_{np} \right)}{\omega_{mn} + \omega_{np}} \left(\frac{f_{np}}{\omega + i\eta - \omega_{pn}} + \frac{f_{nm}}{\omega + i\eta - \omega_{nm}} + \frac{2f_{mp}}{\omega + i\eta - \omega_{pm}} \right) \tag{16}$$

To gain further insights into the relation of the SHG response and the electronic structure of CeAlSi, we investigate here the k space distribution of ξ_{ijk}. As an example, we focus here in particular on ξ_{xxy} in the ferromagnetic state with magnetization $\mathbf{M}_{+x}$ pointing along $\hat{\mathbf{x}} = [110]$. We show in Supplementary Fig. 23a the magnitude of ξ_{xxy} at an incident photon energy of 650 meV. We chose a plane in the Brillouin zone with $k_z = 0.295^{-1}$. In the paramagnetic phase of CeAlSi, this plane contains the W_2 Weyl nodes. In the ferromagnetic phase, the Weyl nodes shift out of the plane (Supplementary Table 7). Red and blue dots therefore indicate the projection of the Weyl nodes onto the plane $k_z = 0.295^{-1}$ in the ferromagnetic phase.

We notice a rather complicated distribution of $|\xi_{xxy}|$ in the plane, but major contributions appear to be related to the Weyl nodes. Note that ξ_{xxy} contains a sum over all bands (Eqs. 15 and 16). The main features of Supplementary Fig. 23a, however, can already be recognized in Supplementary Fig. 23b, where we only consider contributions from electronic transitions between bands -1 and +1 (marked red and blue in Fig. 4c of the main text) [14].

Note that each pair of Weyl nodes is surrounded by two lines of strong contributions to ξ_{xxy}. The inner line corresponds to electronic transitions that are resonant at the incident photon energy (here: 650 meV); the outer line corresponds to transitions resonant at the second-harmonic photon energy (here: 2×650 meV).

As we vary the incident photon energy between 550 meV (Supplementary Fig. 23c) and 950 meV (Supplementary Fig. 23g), we do not observe major changes, but the distance of the inner and outer lines to the Weyl node pair changes. In fact, the distance of the lines of ξ_{xxy} contributions from the Weyl nodes increases approximately linearly with the incident photon energy (dashed black lines in panels c-g), which is due to the linearly dispersive bands in the vicinity of the Weyl nodes.

In Supplementary Fig. 23h, we show a schematic illustration of a possible band structure near the line with $k_{[010]} = 0$, which would qualitatively reproduce our observations from the DFT calculations.

Interestingly, the magnitude of ξ_{xxy} does not change significantly within the considered energy range (in line with the smooth changes of χ_{ijk}, Supplementary Fig. 17). The comparable magnitude of ξ over a wide energy range in combination with smooth changes of the ξ contributions throughout the Brillouin zone rationalize the broadband SHG response that we observed in the experiment.

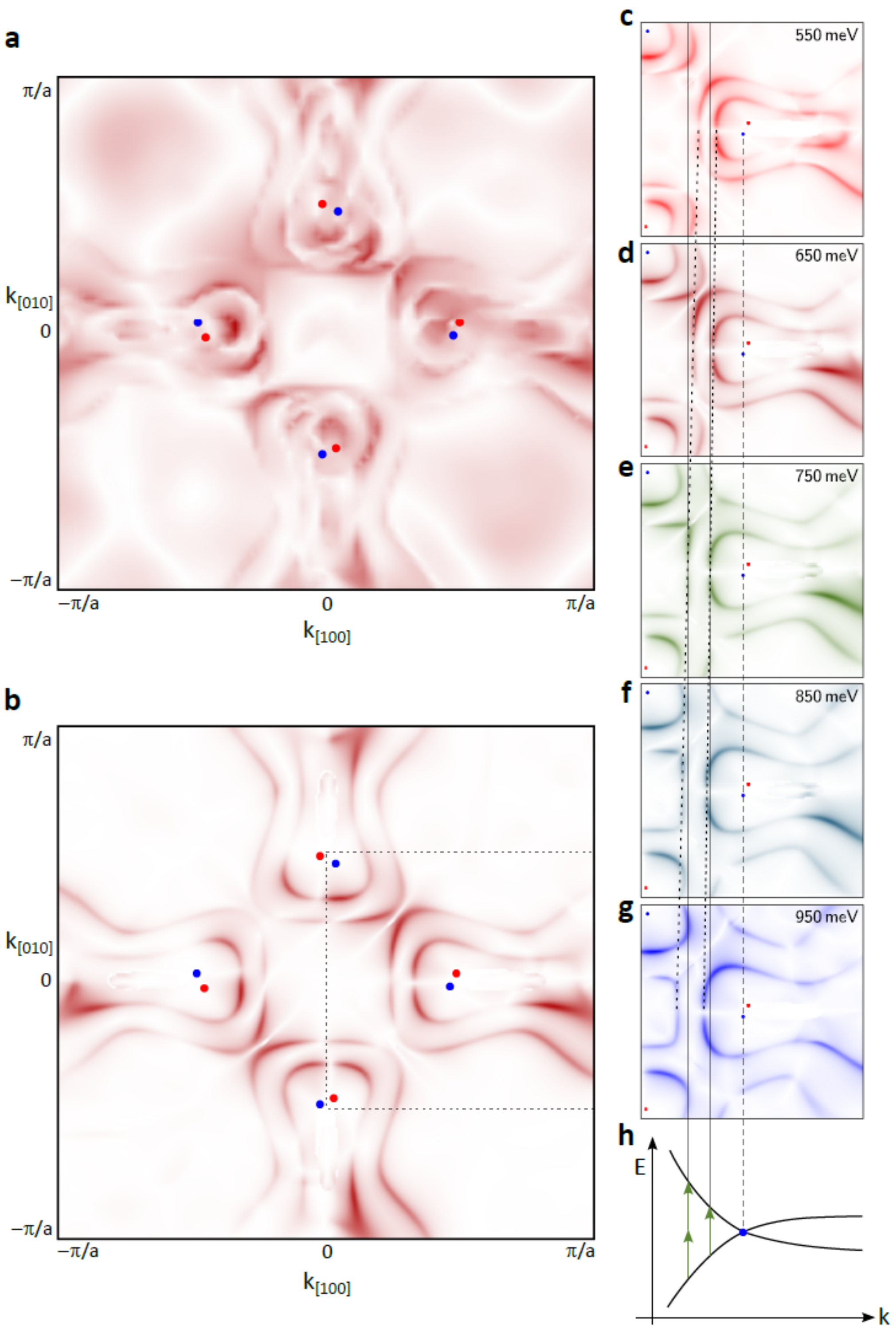

Supplementary Fig. 23. **k space distribution of ξ_{ijk} from first principles. a,** k space distribution of ξ_{xxy} in the plane $k_z = 0.295^{-1}$ for an incident photon energy of 650 meV. Red and blue dots indicate the projection of the W_2 Weyl nodes of different chirality onto the considered plane. **b,** contributions to ξ_{xxy} from electronic transitions between electronic bands -1 and +1. **c-g,** Contributions to ξ_{xxy} at various incident photon energies between 550 meV and 950 meV. Only the section within the dashed box of panel b is shown. **h,** schematic band structure illustrating the origin of the two bands surrounding the Weyl node pair in panels c-g: the closer band corresponds to an electronic transition at the incident photon energy $\hbar\omega$, whereas the outer band corresponds to an electronic transition at $\hbar 2\omega$.

[1] R. Birss, *Symmetry and Magnetism* (North Holland Publishing Company, 1966), second edn.

[2] H.-Y. Yang, *et al.*, Noncollinear ferromagnetic Weyl semimetal with anisotropic anomalous Hall effect, *Phys. Rev. B* **103**, 115143 (2021).

[3] S. Toyoda, M. Fiebig, T. hisa Arima, Y. Tokura, N. Ogawa, Nonreciprocal second harmonic generation in a magnetoelectric material, *Sci. Adv.* **7**, eabe2793 (2021).

[4] J. Mund, *et al.*, Toroidal nonreciprocity of optical second harmonic generation, *Phys. Rev. B* **103**, L180410 (2021).

[5] Y. R. Shen, N. Bloembergen, Interaction between Light Waves and Spin Waves, *Phys. Rev.* **143**, 372 (1966).

[6] Y. Tokura, N. Nagaosa, Nonreciprocal responses from non-centrosymmetric quantum materials, *Nat. Commun.* **9**, 3740 (2018).

[7] V. V. Pavlov, R. V. Pisarev, M. Fiebig, D. Fröhlich, Optical harmonic generation in magnetic garnet epitaxial films near the fundamental absorption edge, *Phys. Solid State* **45**, 662 (2003).

[8] S. Toyoda, *et al.*, Magnetic-field switching of second-harmonic generation in noncentrosymmetric magnet $Eu_2MnSi_2O_7$, *Phys. Rev. Materials* **7**, 024403 (2023).

[9] Y. Sun, *et al.*, Mapping domain-wall topology in the magnetic Weyl semimetal CeAlSi, *Phys. Rev. B* **104**, 235119 (2021).

[10] A. P. Sakhya, *et al.*, Observation of Fermi arcs and Weyl nodes in a noncentrosymmetric magnetic Weyl semimetal, *Phys. Rev. Materials* **7**, L051202 (2023).

[11] D. E. Parker, T. Morimoto, J. Orenstein, J. E. Moore, Diagrammatic approach to nonlinear optical response with application to Weyl semimetals, *Phys. Rev. B* **99**, 045121 (2019).

[12] K. Takasan, T. Morimoto, J. Orenstein, J. E. Moore, Current-induced second harmonic generation in inversion-symmetric Dirac and Weyl semimetals, *Phys. Rev. B* **104**, L161202 (2021).

[13] R.-C. Xiao, *et al.*, Classification of second harmonic generation effect in magnetically ordered materials, *npj Quantum Materials* **8**, 62 (2023).

[14] According to the numerically determined band structure (Fig. 4c in the main text), bands -1 and 0 follow each other closely throughout the Brillouin zone (as do bands +1 and +2). Thus, any of the transitions $-1 \to +1$, $-1 \to +2$, $0 \to +1$, or $0 \to +2$ yields a figure similar to Supplementary Fig. 23b. The choice of bands -1 and +1 for Supplementary Fig. 23b is arbitrary and serves as an example.
