# Supplementary material for: Nonlinear optical diode effect in a magnetic Weyl semimetal
Source: Nat Commun. 2024 Apr 8;15:3017. doi: 10.1038/s41467-024-47291-8 (PMC11271640; doi:10.1038/s41467-024-47291-8)
Supplement: Supplementary file 3 — Description of Additional Supplementary Files [file 41467_2024_47291_MOESM3_ESM.pdf]

<u>Description of Additional Supplementary Files</u>

Supplementary Movie 1 : "Domain imaging using SHG under varying magnetic field along y direction. Polarization: s in/p out; sensitive to M_+y. See Supplementary section 6 for further details."

Supplementary Movie 2 : "Domain imaging using SHG under varying magnetic field along y direction. Polarization: s in/s out; sensitive to M_+/-x. See Supplementary section 6 for further details."

Supplementary Movie 3 : "Operando visualization of domain distribution during the application of electric current. Polarization: s in/p out; sensitive to M_+y."
